# A Configurationally Stable Helical Indenofluorene

**DOI:** 10.1021/acs.orglett.4c02128

**Published:** 2024-07-05

**Authors:** Álvaro Martínez-Pinel, Luis Lezama, Juan M. Cuerva, Raquel Casares, Víctor Blanco, Carlos M. Cruz, Alba Millán

**Affiliations:** † Departamento de Química Orgánica and Unidad de Excelencia de Química Aplicada a Biomedicina y Medioambiente, Facultad de Ciencias, Universidad de Granada, 18071 Granada, Spain; ‡ Departamento de Química Orgánica e Inorgánica, Facultad de Ciencia y Tecnología, Universidad del País Vasco, 48940 Leioa, Spain

## Abstract

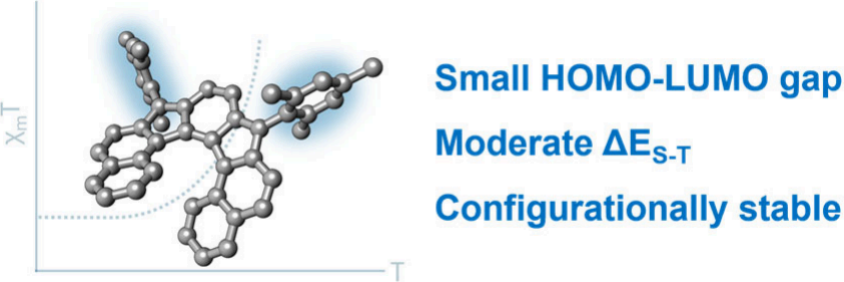

We report the synthesis and study of the optoelectronic, magnetic, and chiroptical properties of a helically chiral diradicaloid based on dibenzoindeno[2,1-*c*]fluorene. The molecule shows a small HOMO–LUMO gap and a moderate singlet–triplet gap, which agrees with the results of DFT calculations. The helical structure of the compound, confirmed by X-ray diffraction, is configurationally stable, which allows the isolation of both enantiomers and the evaluation of the chiroptical properties (ECD).

Indenofluorenes (IFs) are nonalternant conjugated hydrocarbons with alternating fused six- and five-membered rings.^1^ The relative fusion pattern of the rings gives access to a wide family of antiaromatic compounds (Figure 1) with attractive and varied properties.^2−13^ Most of such properties are related with the diradical character provided by their pro-aromatic central quinodimethane unit.^14,15^ Consequently, the IFs need to be stabilized thermodynamically or kinetically to tame the reactivity of the non-bridgehead carbon atom of the five-membered rings (Figure 1, open-shell configurations).^16^ The structural diversity and interesting properties of this class of compounds also invite the exploration of chirality.^17^ The synergy of chirality and unpaired electrons has significant application in molecular optoelectronics and spintronics.^1,18−21^ In this sense, substantial structural modifications have been carried out to provide chiral structures of IF derivatives.^22−28^ Nevertheless, in these examples, the basic 6–5–6–5–6 fused core of indenofluorene is disrupted, and in some cases, low racemization barriers preclude the isolation of enantiopure samples and thus the study of chiroptical properties. Remarkably, the simple indeno[2,1-*c*]fluorene (Figure 1) resembles the structure of a [5]helicene, but it is configurationally unstable.^4−6^ π-Extension of the outermost rings^29^ would enable the exploration of novel helicenoids with enhanced configurational stability, maintaining the basic skeleton of IF. Additionally, such enlargement of the π-system together with the helical distortion should diminish the HOMO–LUMO and the singlet–triplet energy gaps,^30−33^ making these molecules even more appealing for applications.

**Figure 1:**
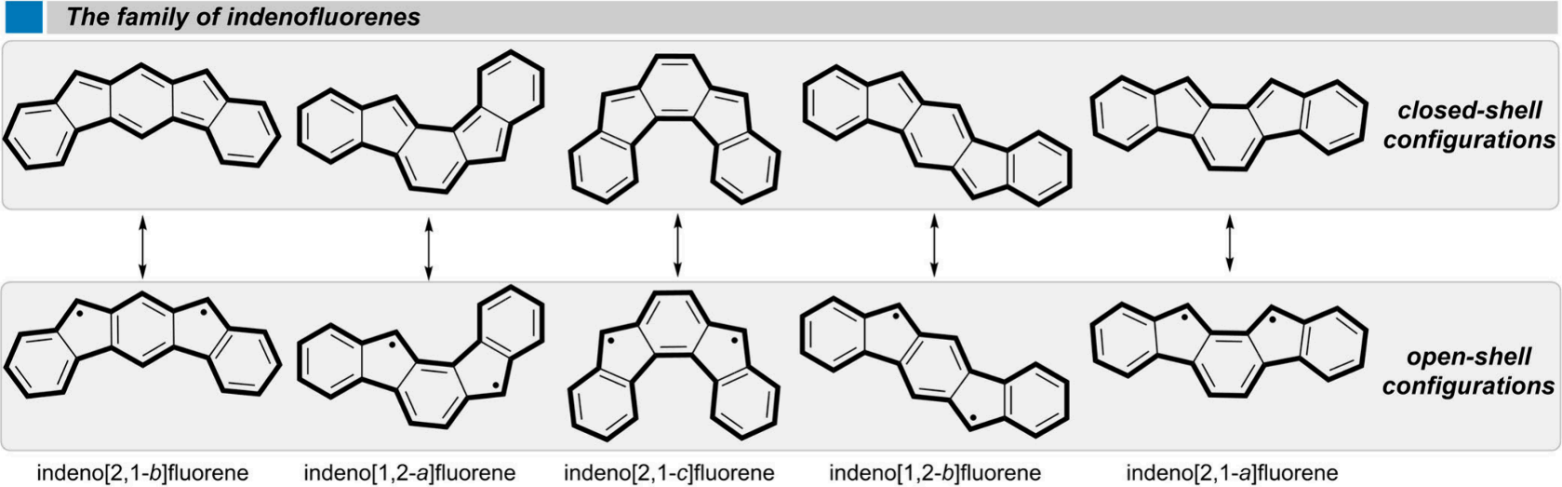
Five regioisomeric indenofluorenes.

Herein we report the synthesis of the [7]helicenoid dibenzoindeno[2,1-*c*]fluorene **IF7H** (Figure 2, right) and the study of its optoelectronic, magnetic, and chiroptical properties. All the experimental data are supported by DFT-theoretical calculations.

**Figure 2:**
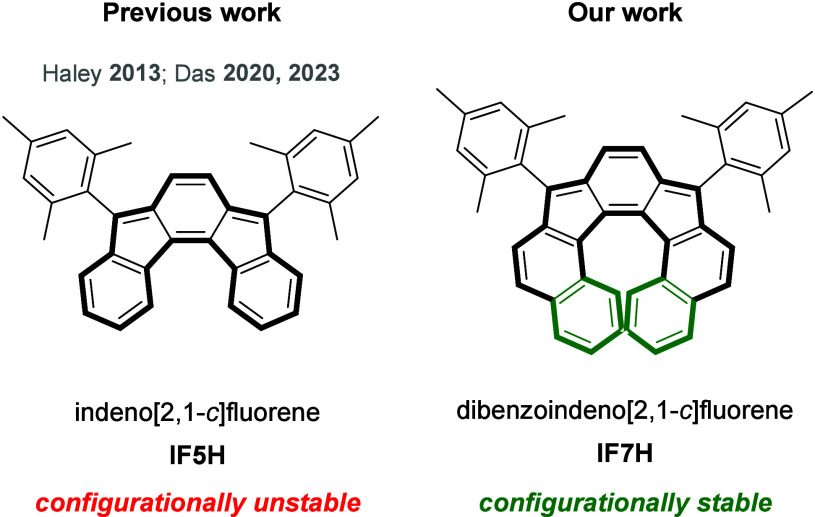
Structure of indeno[2,1-*c*]fluorene **IF5H** (left) and our synthesized extended compound **IF7H** (right).

We carried out the synthesis of **IF7H** starting from diketone **1** (Scheme 1). For the synthesis of the latter, we followed the seven-step synthesis described by Cadart et al. based on an enantioselective [2+2+2] cyclotrimerization of triynes and subsequent oxidation.^34^ The diketone was obtained in 60:40 *e.r*. (*P*:*M* ratio) as reported in the literature. Then we carried out a nucleophilic addition reaction using 2,4,6-trimethylphenylmagnesium bromide to give diol **2** (Scheme 1, see also SI for further details). A final dearomatization reaction using SnCl_2_ gave **IF7H** as a red solid, soluble in toluene or dichloromethane (DCM) among other organic solvents.

**Scheme 1:**
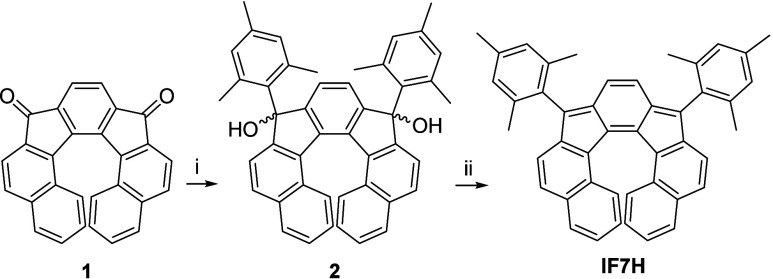
Synthesis of Dibenzoindeno[2,1-*c*]fluorene **IF7H** Conditions: (i) 2,4,6-trimethylphenylmagnesium bromide, tetrahydrofuran (THF), 0 °C, 68%; (ii) SnCl_2_, toluene, 40 °C, 84%.

Compound **IF7H** showed a clear ^1^H NMR spectrum at room temperature with expected signals between 7.6 and 5.7 ppm in the aromatic region (Figure 3a). The signals slightly broaden at high temperature (378 K, 1,1,2,2-tetrachloroethane-*d*_2_,), suggesting the thermal accessibility of the triplet state (see SI). We obtained single crystals of moderate quality of compound **IF7H**, allowing its characterization by X-ray diffraction (Figure 3b–d and S2 in SI). Alternating bond lengths of the central *as*-indacene unit agree with those previously reported for indeno[2,1-*c*]fluorene **IF5H**(4) (Figure 3b). The degree of twisting was analyzed with the sum of the five helicene dihedral angles, resulting in a torsion of 64.4–66.0° (Figure 3c), which is lower than that observed for dinor[7]helicene recently described^23^ (75° on average) but greater than that of **IF5H** (17–30° for three dihedral angles). However, this twisting is not uniformly distributed among all the dihedral angles. Those centered on the five-membered rings are bigger than those centered on the six-membered rings, with the former ranging between 23.8° and 31.5° (mean value 26.7°) and the latter being not larger than 6.0° (mean value 3.9°) (Figure 3c). A similar trend was observed in **IF5H**, where the dihedral angles on the five-membered rings are in the range 8–15°, while the one centered on the six-membered ring was smaller (<6°). This is clearly a different feature from the one observed in [5]- or [7]helicene,^35,36^ which show more homogeneous dihedral angles, ranging from 17° to 28°. Dinor[7]helicene also exhibits a different trend, as the bigger dihedral angle would be the one around the central benzene ring (corresponding to ϕ_3_ in our case, Figure 3c), with a value of ca. 31–35°, while those centered on the five-membered rings are smaller (mainly ca. 4–6°, up to 9°). The interplanar angle between the mean planes of the outer benzene rings was 45.6–46.3°, a similar value to that of [5]helicene (47.3°),^35^ slightly smaller than the interplanar angle exhibited by the dinor[7]helicene (48.6–51.1°) but larger than the corresponding value in the [7]helicene (32.0°) and much larger than that of **IF5H** (10–21°). The distance between the centroids in those two rings was ca. 4.6 Å (Figure 3d), larger than that observed in [7]helicene (3.8 Å)^36^ or the dinor[7]helicene (4.2–4.3 Å), close to that of the [5]helicene (5.0 Å), and smaller than the one in **IF5H** (5.8 Å).

**Figure 3:**
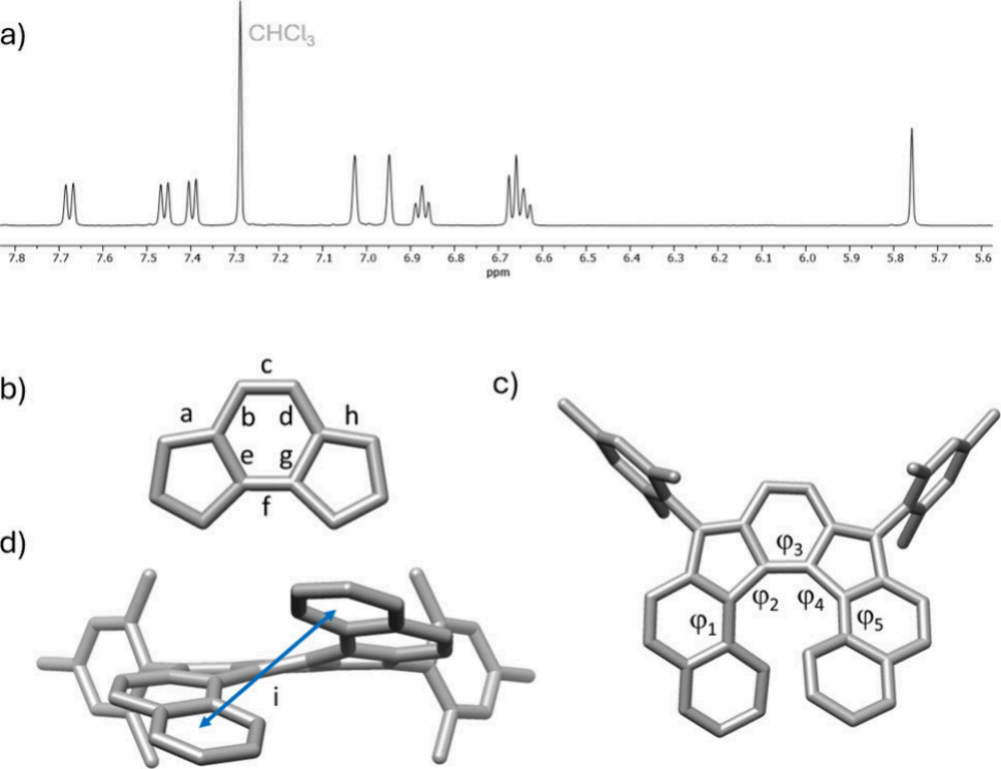
(a) Aromatic region of the ^1^H NMR spectrum of **IF7H**. (b) Bond distances (in Å) in the *as*-indacene core: (a) 1.37–1.38; (b) 1.43; (c) 1.35–1.36; (d) 1.43; (e) 1.48; (f) 1.36–1.38; (g) 1.49; h, 1.37. (c) Top view of the structure showing the dihedral angles (in deg) in the helicene moiety: ϕ_1_, 1.6–6.0; ϕ_2_, 23.8–31.5; ϕ_3_, 4.6–5.6; ϕ_4_, 24.3–27.4; ϕ_5_, 2.5–3.1. (d) Front view showing the distance between the outer helicene rings: (i) 4.61–4.63 Å. H atoms have been omitted for the sake of clarity. Two values are given for some distances and angles, as there are two molecules of **IF7H** in the asymmetric unit.

Then we investigated its photophysical properties (Figure 4). Compound **IF7H** presented an absorption profile spanning from approximately 250 to 1050 nm. The band covering the range 450–550 nm (λ_max_ = 517 nm, ε = 0.60 × 10^4^ M^–1^ cm^–1^) is red-shifted compared to the one in **IF5H** (λ_max_ = 447 nm) due to the π-extension of the system. A broad and weak low-energy band extended to the near-infrared region (λ_max_ = 765 nm, ε = 421 M^–1^ cm^–1^). Again, this band presents a bathochromic shift with respect the lowest energy absorbance in **IF5H**, and it is similar to that observed for angular benzo-fused extended indeno[2,1-*c*]fluorenes.^29^ The latter incorporated triisopropylsilyl (TIPS) acetylene fragments as stabilizing groups for the radical positions, which usually produce a red shift in the absorption compared with the mesityl group. Here, we observed that the different mode of fusion of the extra benzene rings (helical vs angular) can compensate the red-shifted absorption associated with the use of TIPS-acetylene groups. Another interesting feature is the small extinction coefficient for the low-energy band, between three and 10 times lower than for other indeno[2,1-*c*]fluorene derivatives.^4,6,29^ Time-dependent DFT (TDDFT) calculations were carried out at the BS-UB3LYP/6-311G(d) level of theory. The calculated transitions for **IF7H** matched those of the experimental UV–vis. We found a transition at 489 nm (*f* = 0.3306) with a 90% HOMO–1 → LUMO character that matched the band around 500 nm observed in the experimental spectrum. The lowest energy transition was found at 832 nm with a low oscillator strength (*f* = 0.0212), consistent with the low intensity of the band spanning 600–1050 nm. This transition is described with pure HOMO → LUMO character. Thus, the experimental optical energy gap for **IF7H** was 1.20 eV. As for other IF derivatives, compound **IF7H** is nonemissive.^37^

**Figure 4:**
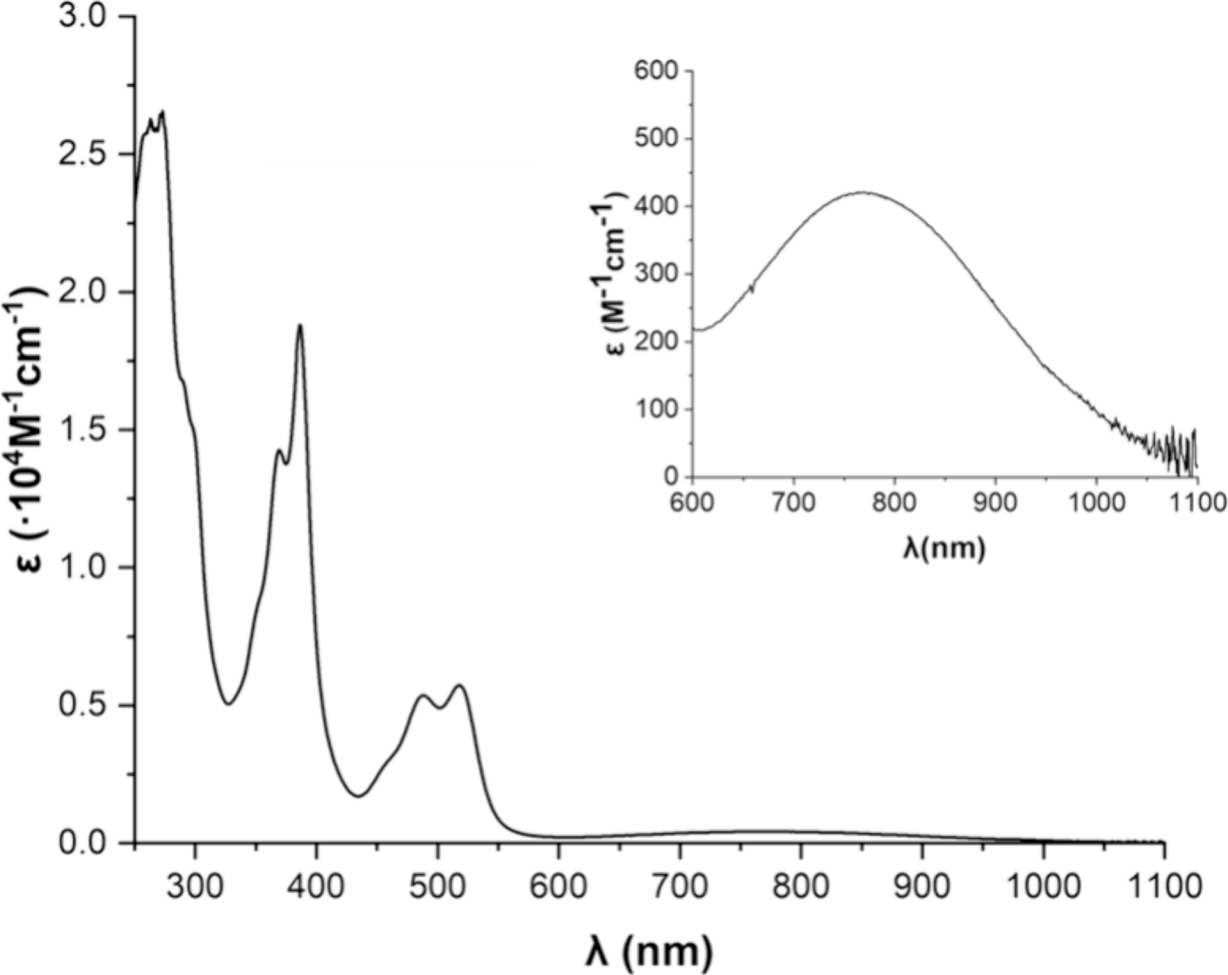
UV–vis spectrum of **IF7H** in CH_2_Cl_2_. Inset shows a magnified view of the 600–1100 nm region.

Then, we carried out cyclic voltammetry (CV) to inspect the electrochemical behavior of **IF7H** (see SI, Figure S3). Compound **IF7H** showed amphoteric redox properties, presenting two quasi-reversible oxidation waves (*E*_1/2_^ox1^ = +0.37 V; *E*_1/2_^ox2^ = +0.85 V) and two quasi-reversible reduction waves (*E*_1/2_^red1^ = −1.38 V; *E*_1/2_^red2^ = −1.85 V). The HOMO energy was estimated at −5.17 eV, ∼0.5 eV higher than that reported for **IF5H**.^4^ The LUMO energy was −3.42 eV. Consequently, the electrochemical HOMO–LUMO gap was estimated to be 1.75 eV, ∼0.3 eV lower than that for **IF5H**. Theoretical calculations agreed with the experimental results, estimating a HOMO–LUMO gap of 1.94 eV.

Next, we investigated its magnetic properties. This compound was active in electron paramagnetic resonance (EPR) (see SI). We observed a featureless and weak peak at room temperature centered at *g* = 2.0025, although we did not observe any half-field signal corresponding to a Δ*m* = ±2 transition featuring the triplet state. In addition, the EPR signal does not vary with temperature. Consequently, we cannot discard the presence of a minor monoradical impurity. We also investigated its magnetic susceptibility with a superconducting quantum interference device (SQUID) magnetometer. The measurements for the powder sample of **IF7H** show a very low and constant value of the χ_m_*T* product (χ_m_ is the molar magnetic susceptibility) between 50 and 300 K and a pronounced increase from 300 K because of the thermal population of the *S* = 1 spin state (Figure 5a). From the Bleaney–Blowers fitting, the singlet–triplet energy gap (Δ*E*_S–T_) was determined to be 9.36 kcal mol^–1^, which agrees with the computed value (10.17 kcal mol^–1^, (BS)-U-LC-BLYP/6-311G(d), see SI). The calculated singlet–triplet gap for **IF5H** was 15.09 kcal mol^–1^, indicating that the extension of the structure, which promotes a more pronounced distortion, lowers the gap considerably. In addition, nucleus independent chemical shift (NICS), harmonic oscillator model of aromaticity (HOMA), and anisotropy of the induced current density (ACID) analyses revealed an increase of the aromatic character in the triplet state of **IF7H**, which contributes to the lowering of the singlet–triplet gap (see SI, Figures S17 and S18).^38^

**Figure 5:**
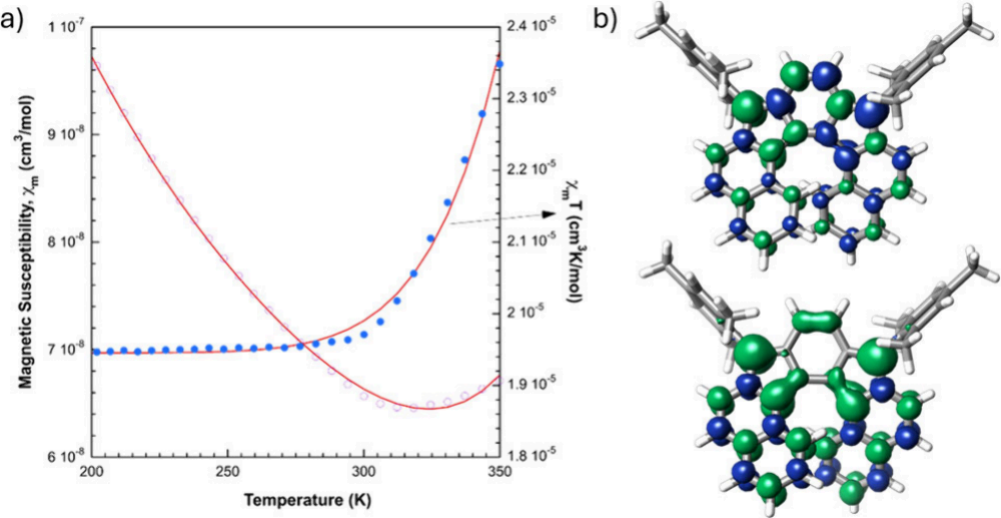
(a) SQUID measurement for **IF7H**. (b) Spin density distribution calculated of the singlet open-shell (top) and triplet (bottom) states of **IF7H** ((BS)-U-LC-BLYP/6-311G(d), isoval = 0.004).

We carried out further theoretical calculations to rationalize the magnetic behavior of our structure, which suggest a singlet open-shell ground state (see SI). Diradical index *y*_0_ is a theoretical parameter that gives an idea of the contribution of the open-shell configuration to the overall structure.^39^ We carried out the calculations using complete active space self-consistent field (CASSCF(2,2) and CASSCF(12,12)), and they indicate a mainly closed-shell configuration for **IF7H** (*y*_0_ = 0.10 and 0.07, respectively). We also performed a natural orbital analysis using different functionals (SI), obtaining similar values, in accordance with the experimental results. These small values are in line with the low magnetic susceptibility values observed in the SQUID experiments. To gain more insight into the spin local distribution, we plotted the spin density for the singlet open-shell and triplet states of **IF7H** ((BS)-U-LC-BLYP/6-311G(d)). The former (Figure 5b, top) presents spin density mainly in the *p*-quinodimethane moiety, while in the latter (Figure 5b, bottom) the spin density is localized around the non-bridgehead five-membered-ring carbon atoms, the central phenyl ring, and the inner part of the helicene. Additionally, some spin density locates in the outer ring.

Finally, we attempted resolution of the enantiomers and investigation of their chiroptical properties. Unfortunately, **IF7H** was not separable by chiral HPLC in any tested conditions. As enantiomers of diketone **1** (60:40 *e.r*.) were also not possible to fully separate by chiral HPLC, we focus on compound **2**. Diastereoisomers were separated by flash column chromatography and further purified by chiral HPLC. Assignation of the *R*/*S* stereocenters was not carried out, as it is inconsequential for the outcome of the final reaction. Finally, after dearomatization using SnCl_2_ the two enantiomers (*P*)-**IF7H** and (*M*)-**IF7H** were obtained (see SI). As shown in Figure 6, the enantiopure samples displayed mirror-image electronic circular dichroism (ECD) spectra with *g*_abs_ values up to 1.2 × 10^–3^ (λ = 308 nm). The absolute configuration of each enantiomer was assigned by TD-DFT. Additionally, configurational stability was studied by calculating the Δ*G*^⧧^_rac_ value. We followed experimentally the decay of the ECD signal over time at different temperatures for (*M*)-**IF7H**. We calculated a Δ*G*^⧧^_rac_ of 24.64 kcal mol^–1^ at 298 K. The racemization rate constant, *k*, allows us to estimate a racemization half-life (*t*_1/2_) of 3.6 days at 298 K. Optimization of the transition state by DFT methods estimated a Δ*G*^⧧^_rac_ of 25.14 kcal mol^–1^ (Figure 6b), in accordance with the evaluated value. The obtained Δ*G*^⧧^_rac_ value is substantially lower than that reported for related compounds.^23,24^ This fact can be explained by the wider fjord region, which favors a less distorted transition state geometry in **IF7H** (see SI, Figures S21 and S22).

**Figure 6:**
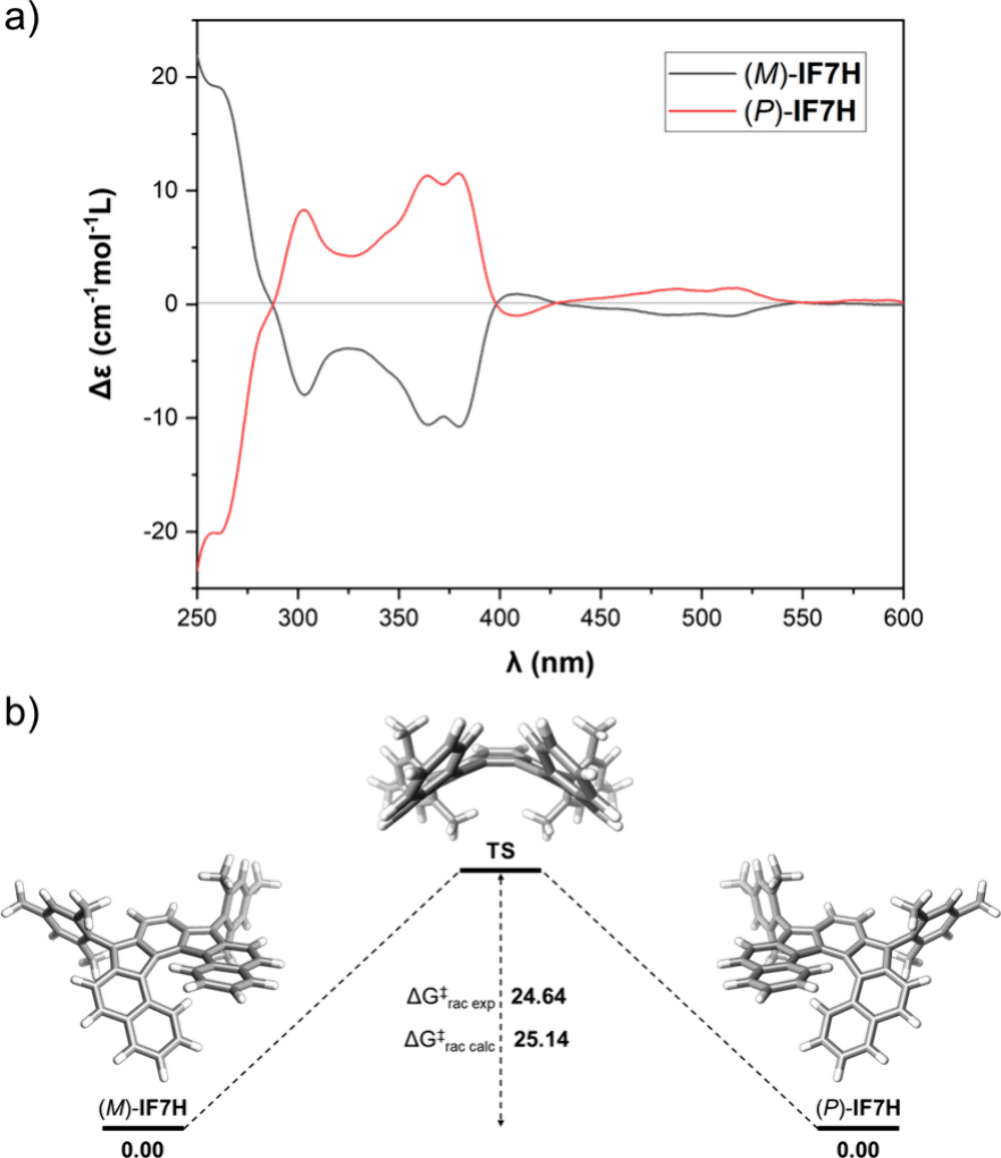
(a) ECD spectra of (*M*)-**IF7H** and (*P*)-**IF7H** in CH_2_Cl_2_. (b) Energy diagram for the isomerization from (*M*)-**IF7H** to (*P*)-**IF7H**; values in kcal mol^–1^.

In conclusion, we have synthesized the simplest configurationally stable helically chiral indenofluorene and evaluated the optoelectronic, magnetic, and chiroptical properties. **IF7H** showed absorption up to ∼1000 nm, a small HOMO–LUMO gap (1.75 eV), and amphoteric character. The molecule presented a mainly closed-shell structure with a small diradical character index and a singlet–triplet gap of 9.36 kcal mol^–1^. The configurational stability of the structure (Δ*G*^⧧^_rac_ = 24.64 kcal mol^–1^ at 298 K) led to the measurement of ECD for each enantiomer. The dissymmetry factor (*g*_abs_) reached 1.2 × 10^–3^. These features, together with its chemical robustness, made this chiral molecule with diradical character suitable for optoelectronic and chiro-spintronic application.^20,21,40^

## Data Availability

The data underlying this study are available in the published article and its Supporting Information.

## Supporting Information Available

Experimental and computational details, synthesis and characterization of compounds, copies of NMR spectra, high-resolution mass spectrum, X-ray details, voltammogram, EPR spectrum, chiral separation details and chromatograms, cartesian coordinates (PDF)
