# Supplementary material for: A Configurationally Stable Helical Indenofluorene
Source: Org Lett. 2024 Jul 5;26(28):6012–7. doi: 10.1021/acs.orglett.4c02128 (PMC11267600; doi:10.1021/acs.orglett.4c02128)
Supplement: Supplementary file 1 — ol4c02128_si_001.pdf [file ol4c02128_si_001.pdf]

SUPPORTING INFORMATION

A Configurationally Stable Helical Indenofluorene

Álvaro Martínez-Pinel,[a] Luis Lezama,[b] Juan M. Cuerva,[a] Raquel Casares,[a] Víctor Blanco,[a] Carlos M. Cruz,[a]* Alba Millán[a]*

[a] Departamento de Química Orgánica and Unidad de Excelencia de Química aplicada a Biomedicina y Medioambiente, Facultad de Ciencias, Universidad de Granada, 18071 Granada, Spain.

[b] Departamento de Química Orgánica e Inorgánica, Facultad de Ciencia y Tecnología, Universidad del País Vasco, 48940 Leioa, Spain.

*email: cmorenoc@ugr.es ; amillan@ugr.es

Table of contents:

1. **General details**……………………………………………………………… S3
2. **Synthetic procedures and characterization of IF7H**…………………………… S4
3. **NMR spectra of compounds**……………………………………………………… S6
4. **High-resolution mass spectrum and theoretical isotopic distribution**…………… S9
5. **Single-crystal X-Ray diffraction**………………………………………………… S10
6. **Cyclic voltammetry**……………………………………………………………… S12
7. **EPR spectra and SQUID measurements** ………………………………………… S13
8. **Studies on enantiopure compounds *P*-IF7H and *M*-IF7H**……………………… S15

   8.1. HPLC separation ……………………………………………………………… S15

   8.2 Dissymmetry factor graphic……………………………………………………… S17

   8.3 Racemization energy barrier…………………………………………………… S18
9. **Computational details**…………………………………………………………… S21
10. **References**………………………………………………………………………… S47

1. General details

Unless otherwise stated, all reagents and solvents were purchased from commercial sources and used without further purification. Anhydrous THF was freshly distilled over Na/benzophenone. Compound **1** was synthesized following the reported procedure.[S1] Mesitylmagnesium bromide is commercially available. Flash column chromatography was carried out using silica gel (40-63 µm) as the stationary phase. Analytical TLC was performed on aluminum sheets coated with silica gel with fluorescent indicator UV254 (Alugram SIL G/UV254, Mackerey-Nagel, Germany) and observed under UV light (254 nm) and/or stained with phosphomolybdic acid (5% methanol solution). Unless otherwise stated, all ^{1}H- and ^{13}C-spectra were recorded on Bruker Avance Neo (400 MHz or 500 MHz) spectrometers at a constant temperature of 298 K. Chemical shifts are reported in ppm and referenced to residual solvent ($CDCl_3$: δ = 7.26 and δ = 77.06 for ^{1}H and ^{13}C respectively). Coupling constants (*J*) are reported in Hertz (Hz). Multiplicities are abbreviated as follow: s = singlet, d = doublet, t =triplet m = multiplet. Assignment of the ^{13}C-NMR multiplicities was accomplished by DEPT techniques. HRMS spectra were obtained using ESI-TOF or GC-EI techniques. IR-ATR spectra were recorded on a Perkin Elmer Spectrum Two IR Spectrometer. Absorption measurements were performed in an Olis DSM172 spectrophotometer using a 1.0 cm path-length quartz cell. The concentration of the solutions for the circular dichroism measurements was $5 \cdot 10^{-5}$ M of the corresponding compound in HPLC grade solvents. The optical HOMO-LUMO energy gap was determined as the intersection of the x axis and a tangent line passing through the inflection point of the lowest-energy absorption.

2. Synthetic procedures and characterization of IF7H

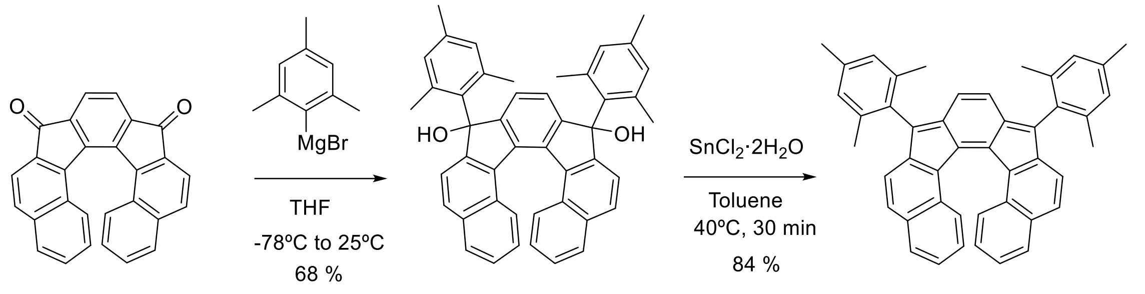

1 **2** **IF7H**

Scheme S1. Strategy followed for the obtention of compound **IF7H.**

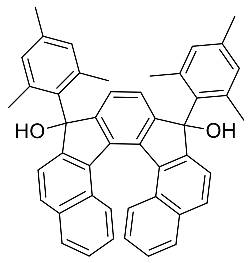

2. Mesitylmagnesium bromide in THF 1M (0.628 mL, 0.628 mmol, 4 equiv.) was added dropwise to a solution of diketone **1** (60 mg, 0.157 mmol, 1 equiv.) in anhydrous THF (13.5 mL) at –78 °C under Ar atmosphere. The reaction mixture was stirred at this temperature for 5 min. Then, it was allowed to warm up to room temperature and was stirred for 30 min. Afterwards, the reaction was quenched with 10% HCl at 0 °C and the product was extracted with EtOAc (3 × 30 mL). The combined organic layers were washed with brine, dried with Na_2SO_4, filtered, and concentrated in vacuo. Column chromatography of the residue on silica gel (Hexane/EtOAc, 7/1) provided the product as a dark orange oil (66 mg, 0.106 mmol, 68 % yield, mixture of isomers).

^{1}H NMR (500 MHz, $CDCl_3$) δ (ppm): 7.89 (d, *J* = 8.2 Hz, 2H), 7.83 (d, *J* = 8.2 Hz, 2H), 7.56 (d, *J* = 8.2 Hz, 2H), 7.44 (s, 2H), 7.23 (d, *J* = 8.5 Hz, 2H), 7.19 (t, *J* = 6.8 Hz, 2H), 7.06 (s, 2H), 6.70 (s, 2H), 6.59 (t, 2H), 3.02 (s, 6H), 2.28 (s, 6H), 1.85 (s, 6H). **^{13}C NMR (126 MHz, $CDCl_3$)** δ (ppm): 152.5, 151.1, 139.3, 136.3, 136.2, 136.1, 135.9, 134.4, 134.0, 132.8, 131.0, 130.4, 129.6, 128.1, 126.9, 125.5, 124.7, 123.4, 121.3, 86.8, 25.9, 22.3, 20.6. **HRMS (ESI) m/z:** $[M+K]^+$ Calcd for $C_{46}H_{38}O_2K$ 661.2509; Found 661.2520. **IR ν_{max} (neat)/cm^{-1}**: 3551, 2954, 2923, 2853, 1705, 1612, 1458, 1378, 1261, 1197, 1113, 1061, 1027, 974, 942, 852, 839, 827, 813, 747, 726, 552, 565.

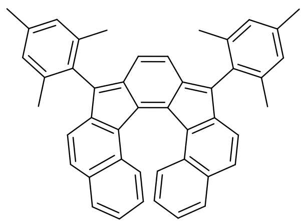

IF7H. Anhydrous and degassed toluene (0.05 M, 2.12 mL) was added under Ar atmosphere to a flask containing diol **2** (66.0 mg, 0.106 mmol, 1 equiv.) and $SnCl_2{\cdot}2H_2O$ (95.7 mg, 0.424 mmol, 4 equiv.) and the mixture was then left to stir at 40 °C in an oil bath for 30 min. After this time, the reaction was diluted with dry CH_2Cl_2 and passed through a short pad of Celite®. The solvent was then removed *in vacuo*. Column chromatography of the residue on neutral alumina (Hexane/CH_2Cl_2, 9/1) provided the product as a red solid (52.4 mg, 0.089 mmol, 84% yield).

^{1}H NMR (500 MHz, CDCl$_3$) δ (ppm): 7.65 (d, *J* = 8.6 Hz, 2H), 7.43 (d, *J* = 8.3 Hz, 2H), 7.37 (d, *J* = 8.2 Hz, 2H), 7.00 (s, 2H), 6.92 (s, 2H), 6.85 (t, *J* = 7.5 Hz, 2H), 6.66-6.60 (m, 4H), 5.73 (s, 2H), 2.46 (s, 6H), 2.34 (s, 6H), 2.07 (s, 6H). **^{13}C NMR (126 MHz, CDCl$_3$)** δ (ppm): 146.1, 144.7, 143.4, 139.3, 136.5, 136.4, 135.8, 135.5, 133.2, 130.0, 129.7, 129.5, 128.7, 127.4, 127.1, 127.1, 124.9, 122.8, 119.3, 119.2, 30.6, 21.6, 13.1. **HRMS (ESI) m/z:** $[M]^+$ Calcd for $C_{46}H_{36}$ 588.2817; Found: 588.2822. **IR ν_{max} (neat)/cm^{-1}**: 2955, 2918, 2850, 1734, 1612, 1514, 1461, 1446, 1378, 1284, 1260, 1194, 1117, 1098, 1026, 850, 819, 803, 759, 744, 720, 639.

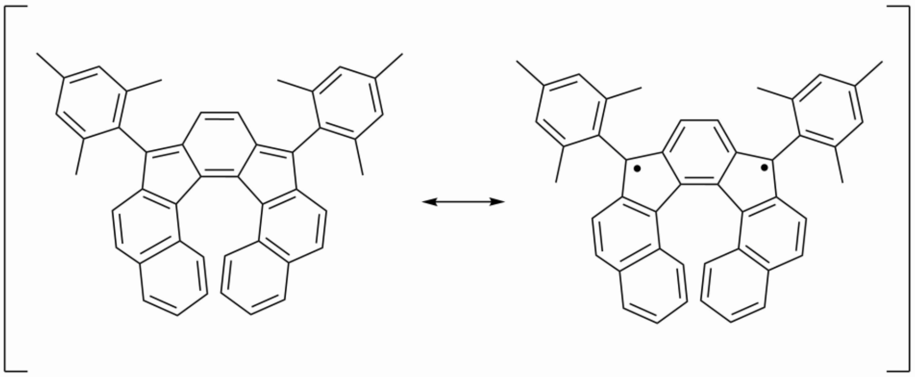

Scheme S2. Quinoidal (left) and diradical (right) resonance representations for compound **IF7H.**

3. NMR spectra of compounds

^{1}H-NMR (400 MHz, $CDCl_3$) and ^{13}C-NMR (126 MHz, $CDCl_3$) of compound 2

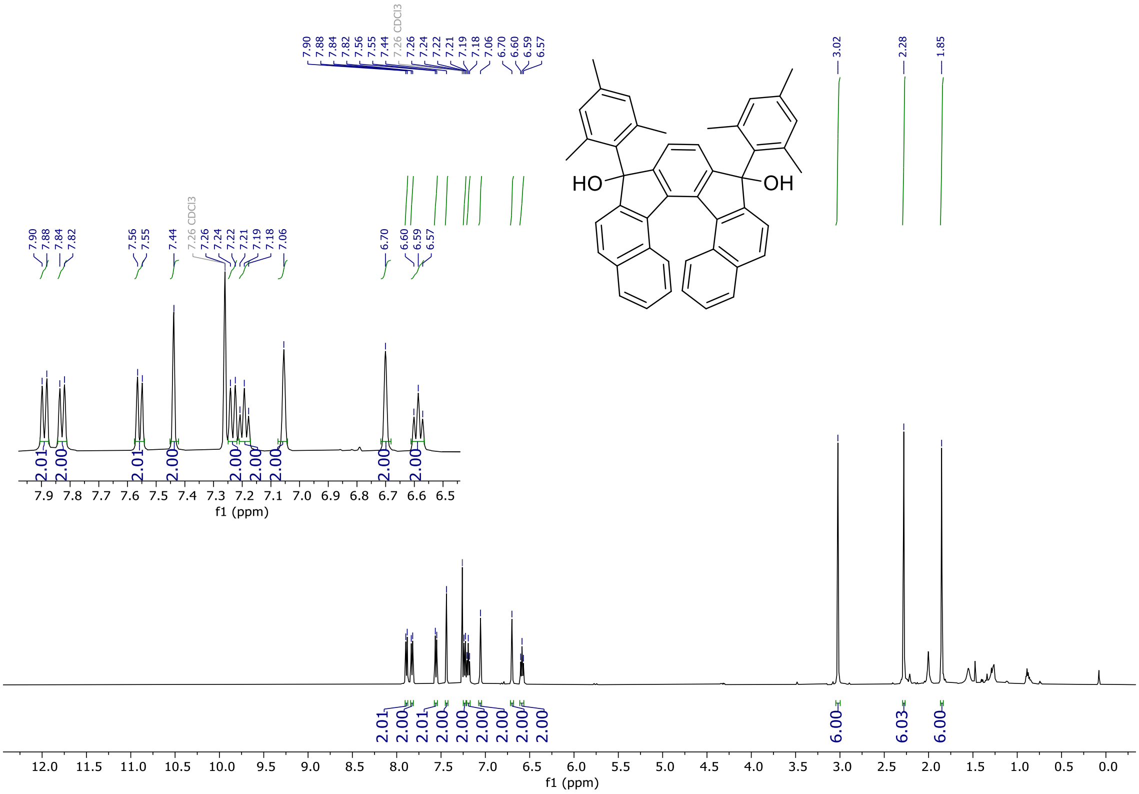

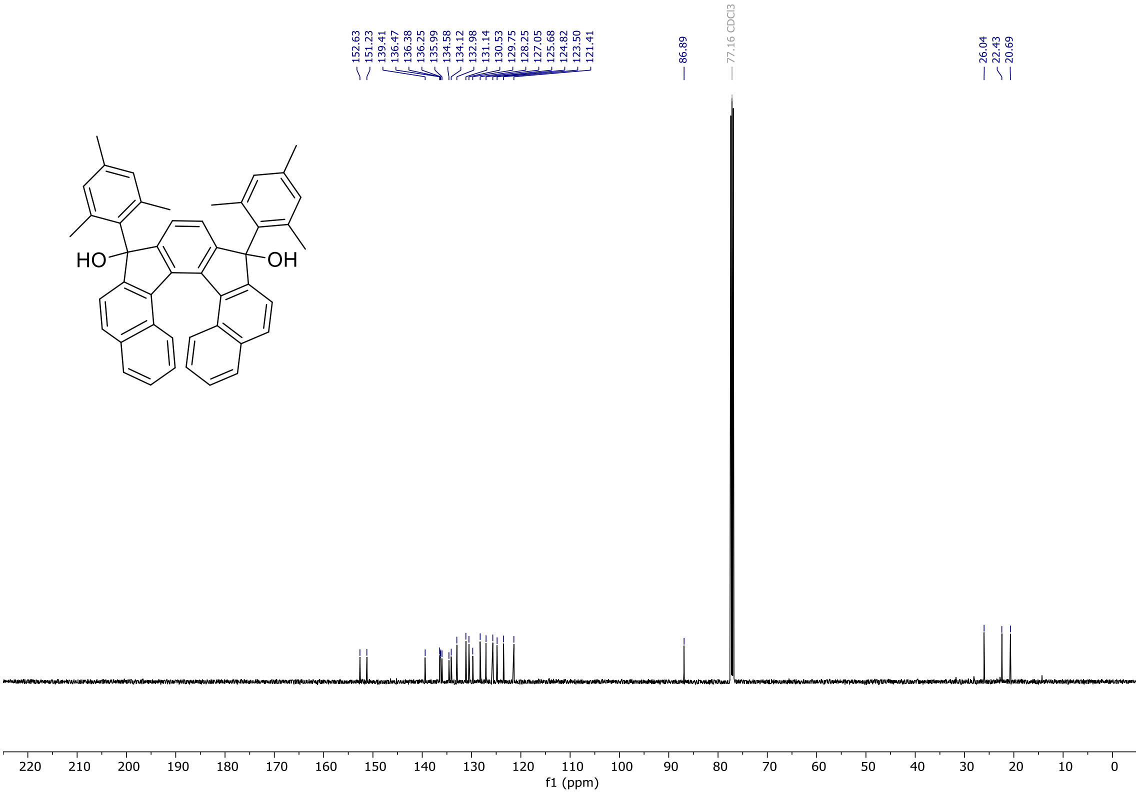

^{1}H-NMR (400 MHz, $CDCl_3$) and ^{13}C-NMR (126 MHz, $CDCl_3$) of compound IF7H

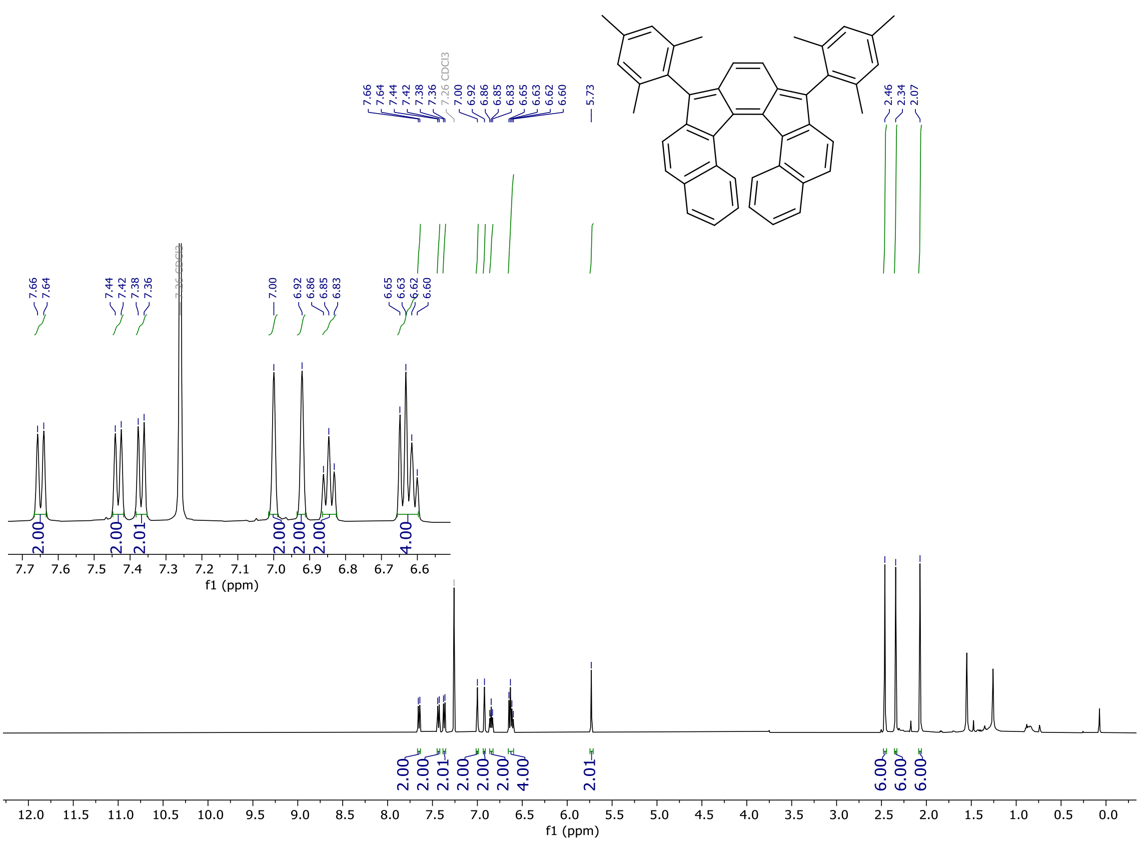

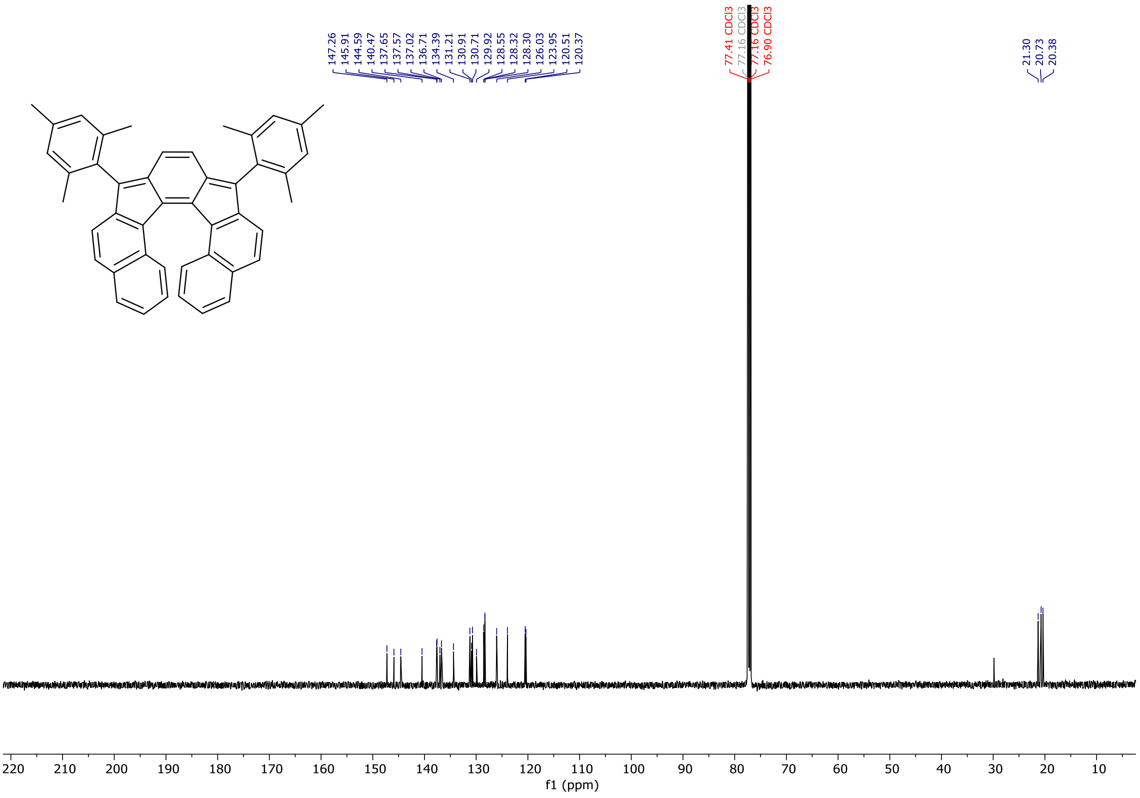

Variable Temperature-^{1}H-NMR experiment (500 MHz, 1,1,2,2-Tetrachloroethane-d_2)

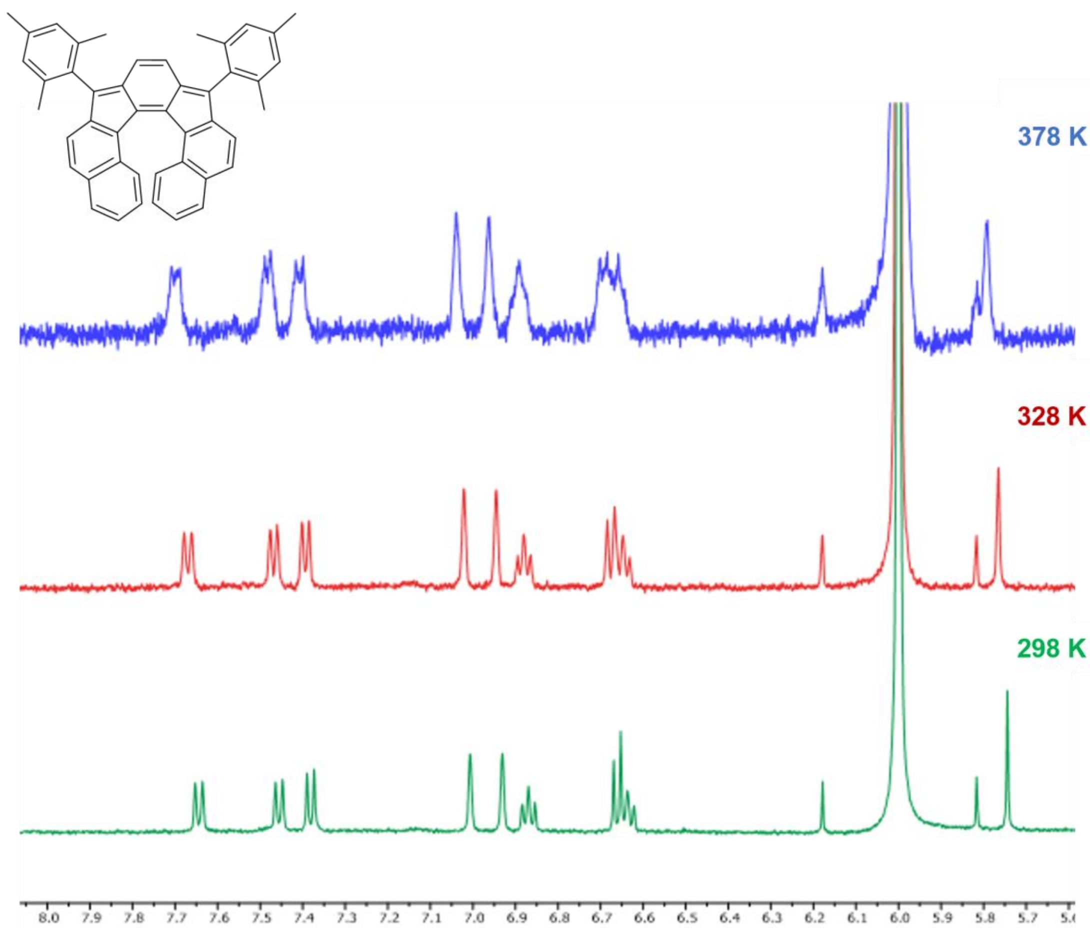

4. High-resolution mass spectrum and theoretical isotopic distribution

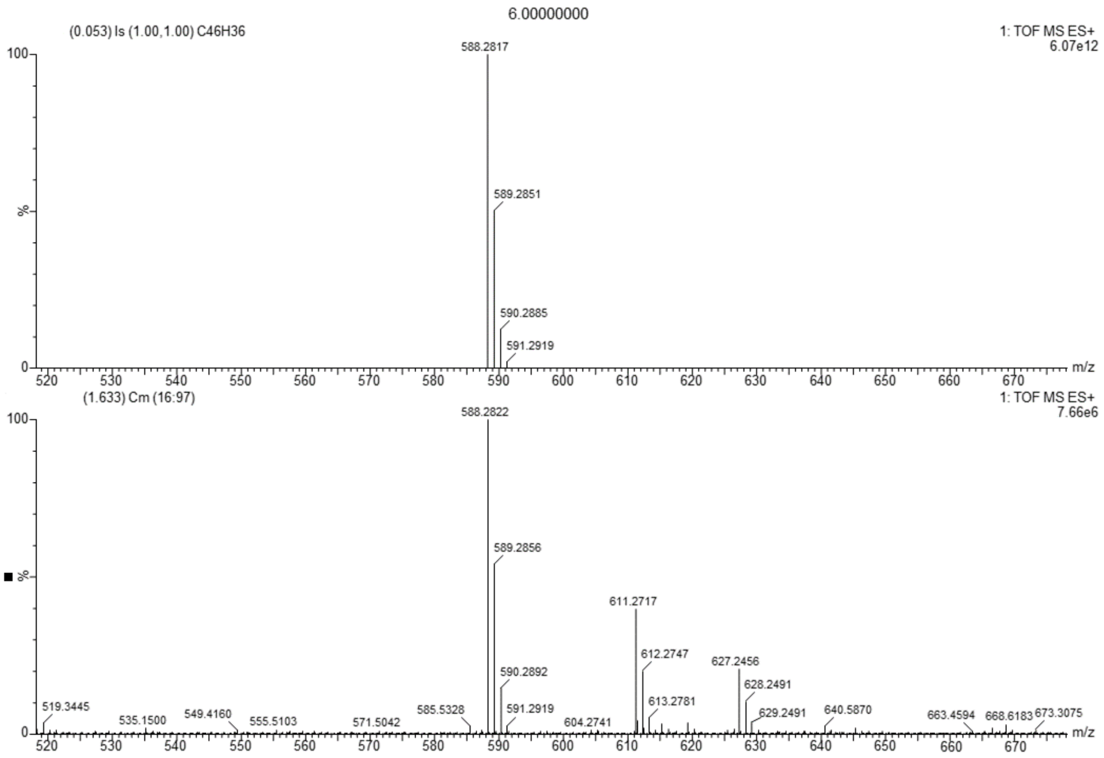

Figure S1. HRMS (ESI) isotopic distribution of the $[M]^+$ peak of compound **IF7H**. Top: Calculated. Bottom: Experimental.

.

5. Single crystal X-Ray diffraction

Single crystals of helical indenofluorene **IF7H** were obtained by slow evaporation of a solution of the compound in ethyl acetate. The crystals obtained were of moderate quality but sufficient for their analysis by X-ray diffraction. A Bruker D8 Venture diffractometer equipped with a Mo radiation source and a PHOTON III detector was used for data collection. The structure was solved with the SHELXT[S2] and refined with SHELX 2019[S3] using the WinGX32[S4] graphical interface. The refinement was carried out with the full-matrix least-squares against F^2 procedure. C–H hydrogen atoms were placed in idealized positions (U_{eg}(H) = 1.2U_{eg}(C) or U_{eg}(H) = 1.5U_{eg}(C)) and were allowed to ride on their parent atoms.

The crystal analyzed displayed twinning and it was refined as a 2-component non-merohedral twin. The components were identified upon data processing and a hklf5 file generated. Solution and initial refinement were performed using the hklf4 reflection data file and a final refinement was carried out with the hklf5 file.

Moreover, although a full set of data was collected, at very high angle only noise was mainly observed due to the moderate quality of the crystal and these data was omitted for the refinement.

X-ray diffraction measurement and refinement data for **IF7H**: Chemical formula, $C_{46}H_{36}$; *Mr*, 588.75; crystal size [mm^3], 0.616 x 0.217 x 0.024; temperature, 100(2) K; wavelength [Å], 0.71073 (Mo Kα), crystal system, triclinic; space group, *P*-1; *a* [Å], 11.509(3); *b* [Å], 17.723(4); *c* [Å], 18.235(4); α [°], 62.211(4); β [°], 80.221(7); γ [°], 89.974(7); *V* [Å^3], 3229.5(12); *Z*, 4; ρ_{calcd} [mg m^{-3}], 1.211; μ [mm^{-1}], 0.068; F(000), 1248; ϑ range [°], 2.004 to 20.980; *hkl* ranges, −11/11, −15/17, 0/18; reflections collected, 53225; independent reflections, 6614; completeness to ϑ = 20.980°, 88.7% (from SHELX), 95.5% (from checkCIF/PLATON); absorption correction, semi-empirical from equivalents; refinement method; full-matrix least-squares on F^2 ; Final *R* indices [$I>2\sigma(I)$], R_1 = 0.0496, wR_2 = 0.0989; *R* indices (all data), R_1 = 0.0907, wR_2 = 0.1167; goodness-of-fit on F^2, 1.052.

Deposition Number CCDC 2323643 contains the supplementary crystallographic data for this paper. These data are provided free of charge by the joint Cambridge Crystallographic Data Centre and Fachinformationszentrum Karlsruhe Access Structures service.

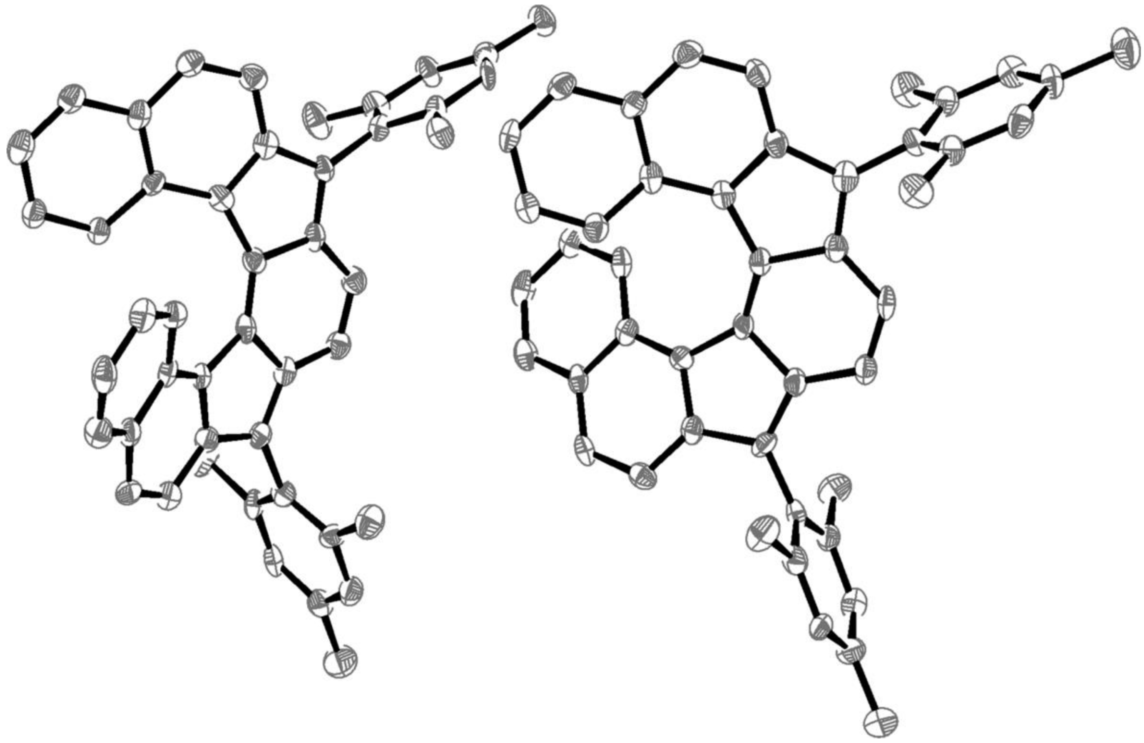

Figure S2. ORTEP drawing of the two molecules of **IF7H** present in the asymmetric unit. The thermal ellipsoids are shown at 50% probability.

6. Cyclic voltammetry

Cyclic Voltammetry (CV) were carried out on a PGSTAT2014 potentiostat/galvanostat (Metrohm Autolab B. V.) with a three-electrode cell under Ar atmosphere at 25 °C. A Pt-wire counterelectrode, an Ag wire quasireference electrode and a glassy carbon disk working electrode were used. CH_2Cl_2 was used as solvent to prepare a 0.1 M solution of tetra-*n*-butylammonium hexafluorophosphate ($TBAPF_6$) which was used as work solution. The Pt wire counter electrode and Ag wire quasi-reference electrode were cleaned by flame treatment. The glassy carbon disc working electrode was polished according to the literature.[S5] A 1.5 mM solution of **IF7H** in CH_2Cl_2 was used. The scan rate was 0.05 V/s. The starting potential was 0 V against Ag reference electrode. Potential values are referred to ferrocenium/ferrocene (Fc^+/Fc). Fc added as an internal reference after each measurement. Potential values are reported in V *vs.* Fc^+/Fc. HOMO and LUMO energies were estimated from first oxidation/reduction half-wave potentials assuming an absolute ionization energy of 4.8 eV for ferrocene. We followed the IUPAC convention for CV plotting.

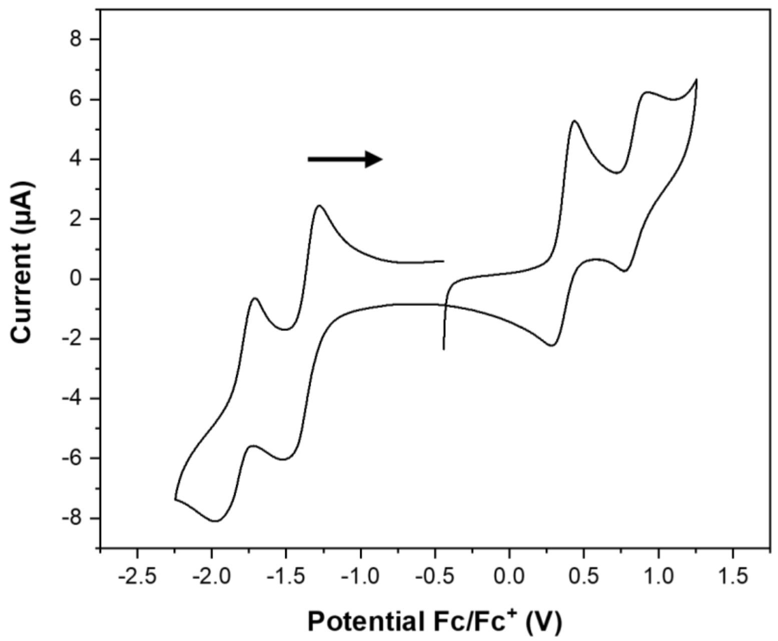

Figure S3. Voltammogram of **IF7H** in CH_2Cl_2. The arrow indicates the direction of the scan.

7. EPR spectra and SQUID measurements

Experimental

X-band EPR measurements were carried out on a Bruker ELEXSYS 500 spectrometer equipped with a super-high-Q resonator ER-4123-SHQ, standard Oxford Instruments low-temperature devices and a Bruker ER4131VT temperature controller for liquid and gas nitrogen. The magnetic field was calibrated by a NMR probe and the frequency inside the cavity was determined with a Hewlett-Packard 5352B microwave frequency counter. Data were collected and processed using the Bruker Xepr suite.

Temperature dependent magnetic measurements were performed between 5 and 350 K with applied field of 1T using a MPMS3 SQUID magnetometer (Quantum Design). The experimental susceptibilities were corrected for the diamagnetism of the constituent atoms, by using Pascal tables, and the sample holder.

EPR spectra

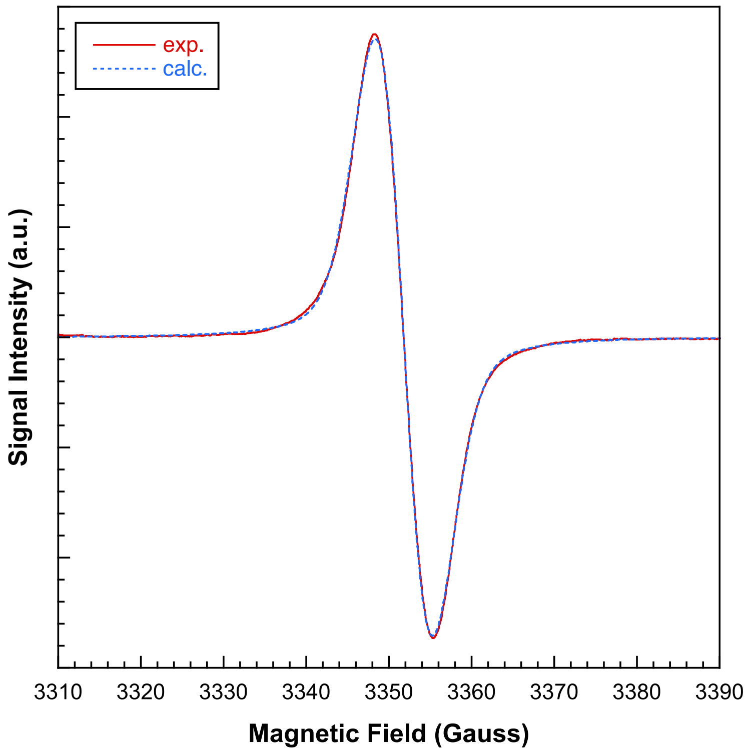

Figure S4. X-band EPR spectrum recorded at room temperature on a solid sample of **IF7H**. Signal remains essentially unchanged between 5 and 380 K. The best fit was obtained with g=2.0025 and ΔH_{pp}=6.9 Gauss.

Magnetic Measurements

The thermal evolution of the magnetic molar susceptibility, χ_m, and the $\chi_m T$ product of compound **IF7H** were fitted to the Bleaney-Bowers equation[S6] for the magnetic susceptibility of isotropically coupled dinuclear S=1/2 systems adding an impurity term (δ) defined as the molar fraction of noncoupled S=1/2 species.

$$\chi_m = (1-\delta)\frac{2Ng^2\beta^2}{kT(3+\exp(-2J/kT))} + \delta\frac{Ng^2\beta^2}{3kT}$$

where the singlet-triplet energy gap (*2J*) is defined by the Hamiltonian $H = -2J \cdot S_1 \cdot S_2$ ($S_1=S_2=1/2$); *g* is the Lande's g factor; *N*, β and *k* are the Avogadro's number, the Bohr magneton, and Boltzmann's constant, respectively. The best-fit parameters obtained by minimizing the reliability factor $R = \Sigma[(\chi_m)_{exp}-(\chi_m)_{cal}]^2/\Sigma[(\chi_m)_{exp}]^2$ are g = 2.01, J = -1637 cm^{-1} (ΔE=9.36 kcal/mol), δ=5.2e^{-5} and R = 4.6x10^{-4}.

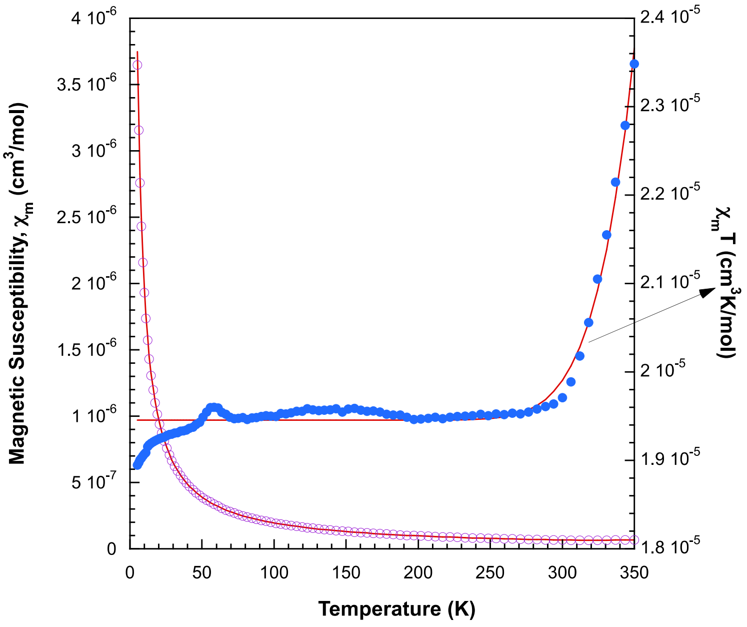

Figure S5. Magnetic behavior of **IF7H** from 4-350 K. The solid lines represent the best fits to the Bleaney-Bowers equation.

8. Studies on enantiopure compounds *P*-IF7H and *M*-IF7H

8.1. HPLC separation

HPLC analysis were performed on an Agilent Serie 1260 apparatus (quaternary pump, auto sampler, column thermostat and diode array detector) using a CHIRALPAK® IA column (250 x 10mm, 5µm) packed with silica gel surface covalent bond with amylose-tris (3,5-dimethylphenylcarbamate and HPLC grade solvents. The column temperature was set at 25 °C.

We first attempted the separation **IF7H**. However, we could not get any separation.

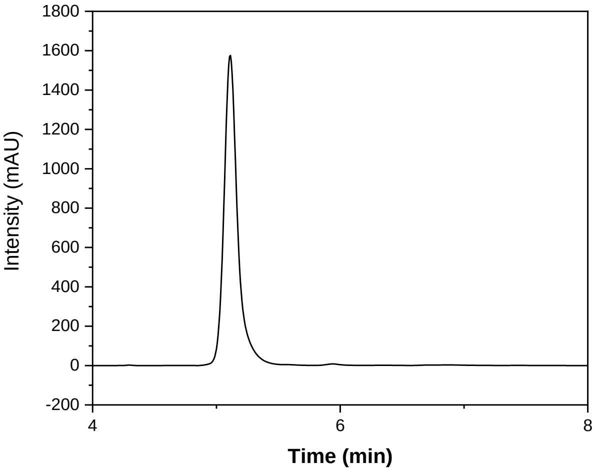

Figure S6. Example of chiral HPLC chromatogram of **IF7H**. HPLC Conditions: IA, hexane/ dichloromethane = 80/20, flow rate = 3.8 mL/min, λ= 384 nm, t_R = 5.11 min. No separation was obtained in any other tested conditions.

We then try to separate the diketone **1**. Although we could partially separate the enantiomers, the uncomplete separation precludes an efficient collection of enantiopure samples.

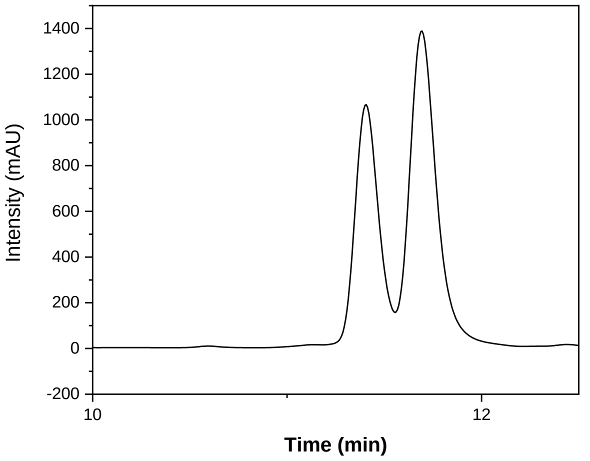

Figure S7. Example of chiral HPLC chromatogram of **1** (40:60 *M*:*P* ratio). HPLC Conditions: IA, hexane/ dichloromethane = 85/15, flow rate = 3.8 mL/min, λ = 387 nm, t_R = 11.4 min (minor), 11.7 min (major).

Finally, we followed the strategy depicted in Scheme S3 for the isolation of *P*-**IF7H** and *M*-**IF7H**. Firstly, the diastereoisomers of **2** were separated by flash column chromatography (FCC), and further separated by chiral HPLC. In these cases, the dearomatization reaction was done at room temperature to avoid racemization.

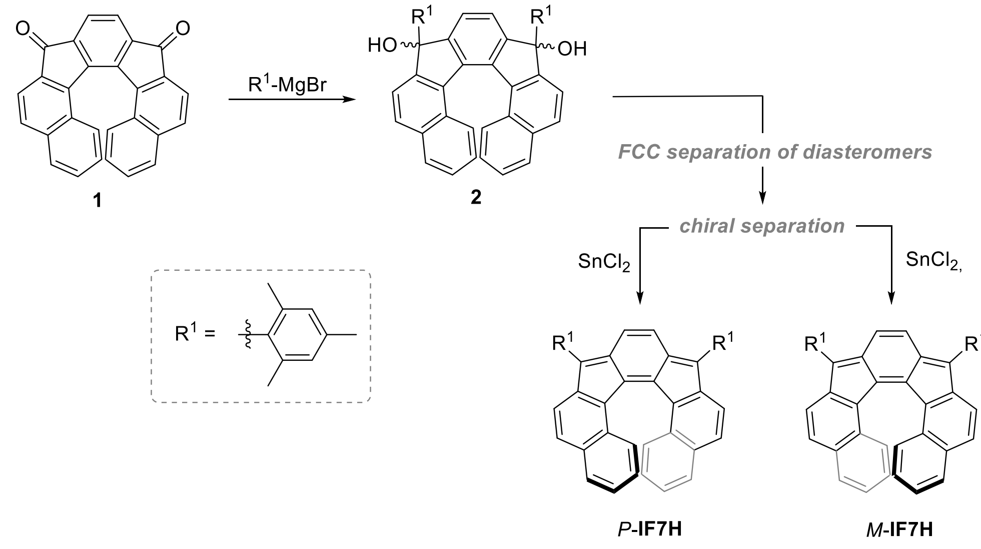

Scheme S3. Strategy followed for the obtention of enantiopure **IF7H**.

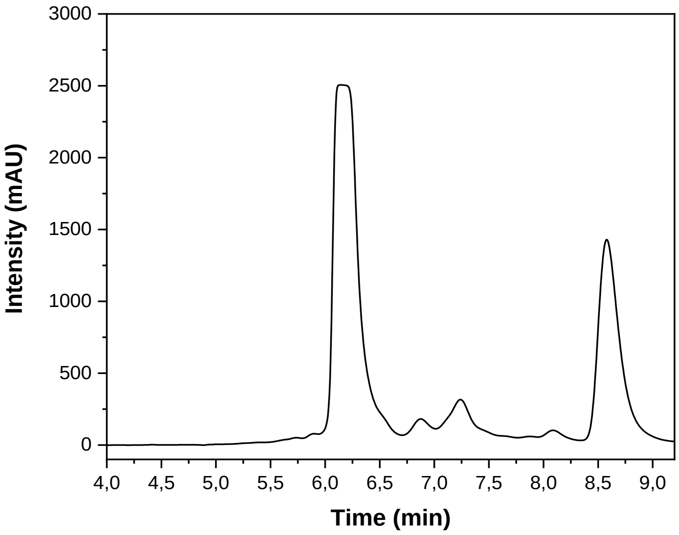

Figure S8. Chiral HPLC chromatogram of **2**. HPLC Conditions: (IA, hexane/ ethyl acetate = 80/20, flow rate = 3.8 mL/min, λ = 386 nm, t_R = 6.1 min (major), 8.9 min (minor). Volume and concentration of injected sample: 1000 µL of 2 mg/mL in hexane / ethyl acetate 90/10.

8.2. Dissymmetry factor graphics

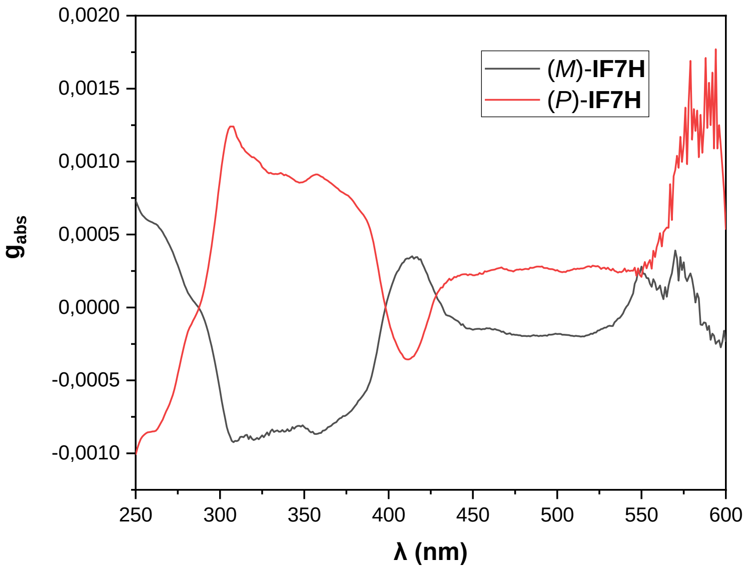

Figure S9. Dissymmetry factor of **IF7H** in DCM in the range 250-600 nm.

8.3. Racemization energy barrier

The decay of the enantiomeric excess in ECD of compound (*M*)-**IF7H** was measured in toluene at five temperatures (40, 50, 60, 70 and 80 °C) in an Olis DSM172 spectrophotometer with peltier and using a 1.0 cm path-length quartz cell.

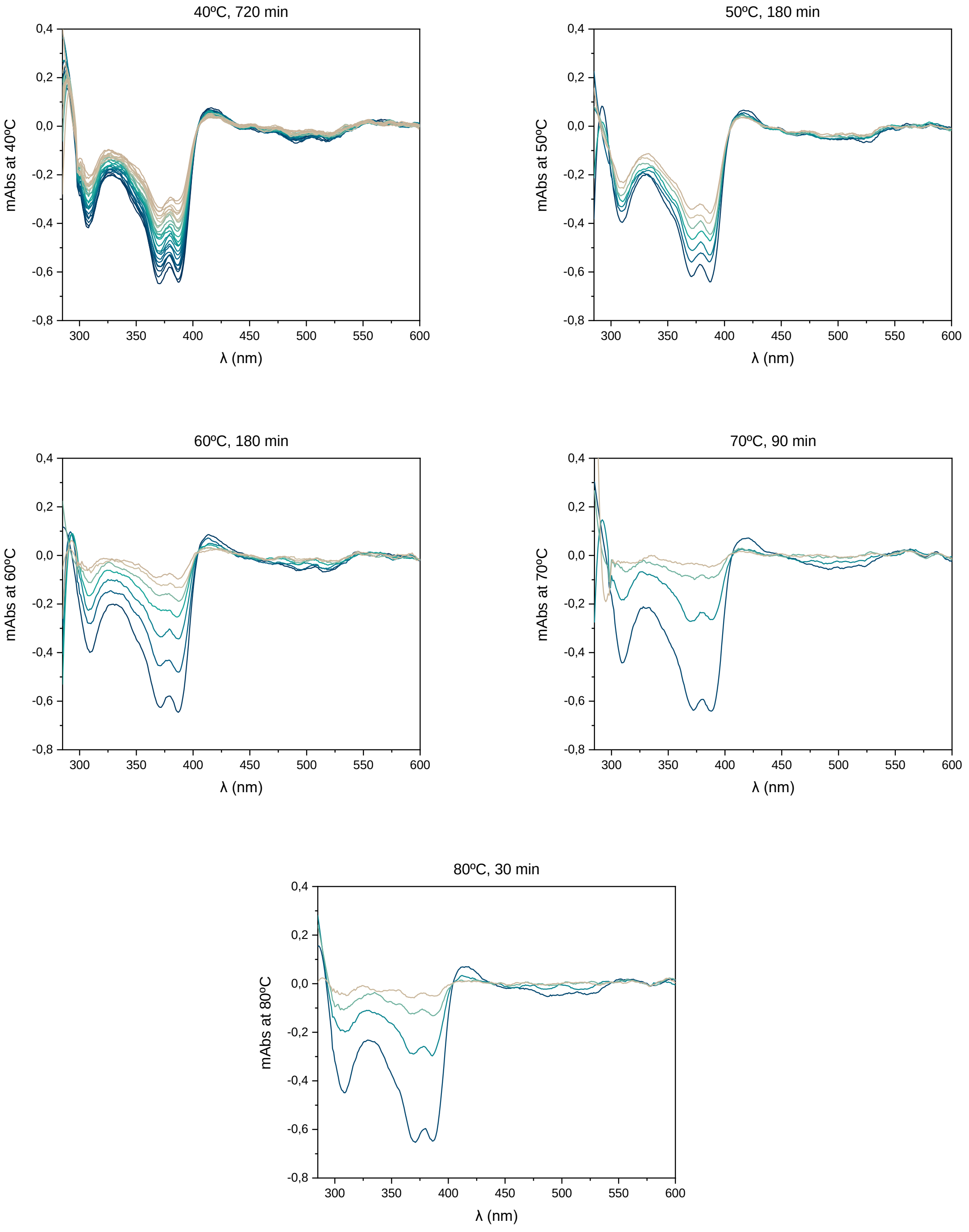

Figure S10. Decay of the enantiomeric excess of (*M*)-**IF7H** in toluene at different temperatures followed by ECD.

To estimate the value of $\Delta G^{\ddagger}$, the values of $Ln(A_t/A_0)$ at each temperature were plotted, where A_t is the value of CD at a given time at 388 nm and A_0 is the initial value of CD at 388 nm, and fitted to a first-order velocity constant k (s^{-1}) (Equation 1 and Figure S11, left).

$$Ln(A_t/A_0) = -kt$$

Equation 1.

Once the kinetic constants for each temperature have been obtained, the racemization half-life $t_{1/2}$ and the activation energy E_a can be determined. The racemization half-life, $t_{1/2}$ (s), can be achieved using the Equation 2. The activation energies, E_a (kcal mol^{-1}), can be calculated using the linear Arrenhius equation (Equation 3 and Figure S11, right) where R is the ideal gas constant ($1.98 \cdot 10^{-3}$ kcal mol^{-1} K^{-1}).

$$t_{1/2} = \frac{ln\ 2}{k}$$

Equation 2.

$$Ln\ k = -\frac{E_a}{R} \cdot \frac{1}{T}$$

Equation 3.

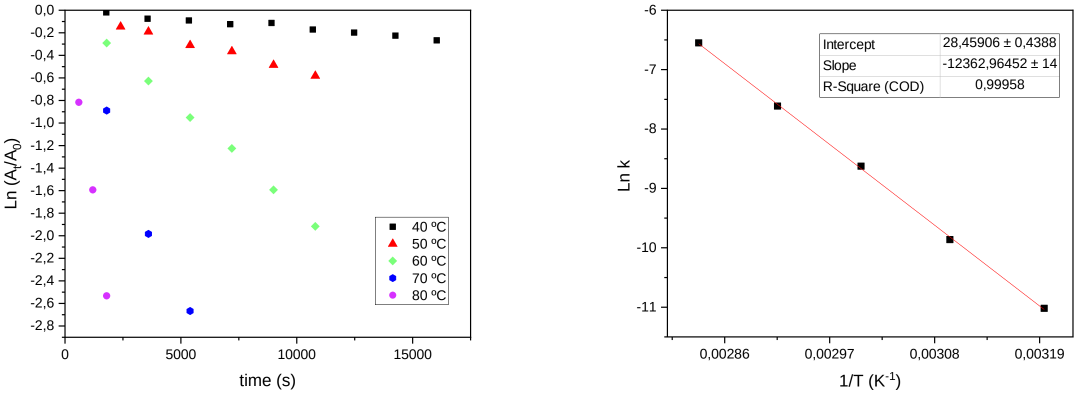

Figure S11. Decay of the ECD signal of (*M*)-**IF7H** with time at 388 nm at different temperatures (left) and Arrhenius representation for the racemization of (*M*)-**IF7H** (right).

Using the kinetic racemization constants in the linear Eyring-Polanyi equation (Equation 4) the thermodynamic parameters $\Delta H^{\ddagger}$ and $\Delta S^{\ddagger}$ can be determined (Figure S12).

$$\ln\frac{k}{T} = -\frac{\Delta H^{\ddagger}}{R} \cdot \frac{1}{T} + Ln\frac{\kappa k_B}{h} + \frac{\Delta S^{\ddagger}}{R}$$

Equation 4.

k_B is the Boltzman constant ($1.380662 \cdot 10^{-23}$ J K^{-1}), h the Planck constant ($6.626176 \cdot 10^{-34}$ J s), R the ideal gas constant ($1.98 \cdot 10^{-3}$ kcal mol^{-1} K^{-1}), $\Delta H^{\ddagger}$ is the enthalpy of activation, $\Delta S^{\ddagger}$ is the entropy of activation, T is the temperature and κ (0.5) is the transmission coefficient.

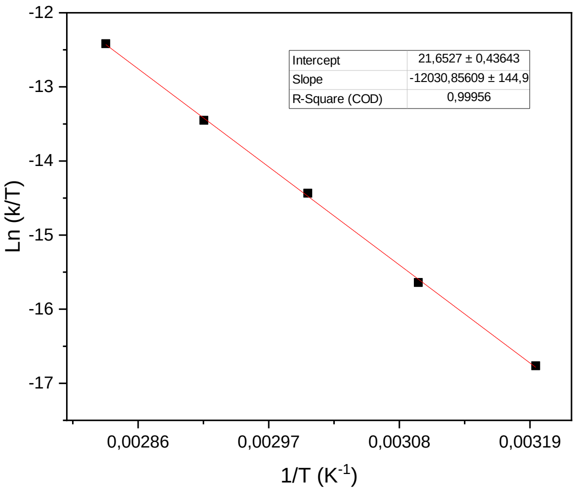

Figure S12. Eyring-Polanyi representation for the racemization of (*M*)-**IF7H**.

Finally, from the Gibbs equation (Equation 5) the value of $\Delta G^{\ddagger}$ is obtained.

$$\Delta G^{\ddagger} = \Delta H^{\ddagger} - T \cdot \Delta S^{\ddagger}$$

Equation 5.

Table S1. Kinetic and thermodynamic data

Kinetic Data				***Thermodynamic Data***	
**Temperature (°C)**	**k (s^{-1})**	**$t_{1/2}$ (min)**	**E_a (kcal mol^{-1})**	***ΔH‡* (kcal mol^{-1})**	23.82
25 (*)	$2.20 \cdot 10^{-6}$	5252.9	24.47		
40	$1.64 \cdot 10^{-5}$	704.4			
50	$5.21 \cdot 10^{-5}$	221.7		***ΔS‡* (kcal mol^{-1} K^{-1})**	$-2.77 \cdot 10^{-3}$
60	$17.93 \cdot 10^{-5}$	64.4			
70	$49.37 \cdot 10^{-5}$	23.4		***ΔG‡* (kcal mol^{-1})**	24.64
80	$14.30 \cdot 10^{-4}$	8.1			

(*) The racemization kinetic constant, k, and the racemization half-life time, $t_{1/2}$, at 25 °C have been determined by extrapolation using the regression line shown in Figures S11, right and Equation 2 respectively.

9. Computational details

The Gaussian 09[S7] (Revision B.01), Gaussian 16[S8] (Revision C.02) and Orca[S9] (version 5.0.2) software packages were used to investigate the structural and electronic properties of compound **IF7H**. DFT geometry optimizations were performed using the restricted version of the Becke three-parameters exchange function in combination with the Lee-Yang-Parr correlation functional (RB3LYP). The basis set 6-311G(d) was used for the optimizations. Solvent effects were included via the Polarizable Continuum Model (IEFPCM) as implemented in Gaussian 09 with the dielectric constant of dichloromethane. Optimized geometries were confirmed to be stationary points by analysis of their vibrational frequencies. The stability of the DFT wavefunction was tested over each optimized geometry and optimized if any restricted→unrestricted instability was found. In all cases, the structures were reoptimized using the unrestricted version of the B3LYP functional (UB3LYP), with the 6-311G(d) basis set and considering dichloromethane as solvent, using the broken symmetry formalism (BS) for the singlet open-shell calculations and the transition state geometry. Calculation of the closed-open-shell and singlet-triplet gap energies were performed using different functionals (B3LYP, CAM-B3LYP, LC-BLYP and M06-2X), with the 6311G(d) basis set in all cases. The singlet-triplet gap was computed from the equation:

$$\Delta E_{S-T} = (E_{OS} - E_T)\frac{\langle S^2\rangle_T}{\langle S^2\rangle_T - \langle S^2\rangle_{OS}}$$

Aromaticity and ring current indexes were studied. We computed the nucleous independent chemical shift (NICS) at the centroid of each ring ($NICS(0)_{iso}$) at the (BS)-U-LC-BLYP/6311G(d) level of theory. The harmonic oscillator model of aromaticity (HOMA) was calculated from the optimized geometries. The anisotropy of the induced current density (ACID) maps were generated using the (BS)-U-LC-BLYP/6311G(d) level of theory using Gaussian 16.

The 50 lowest electronic transitions were calculated by TD-DFT as implemented in Gaussian 09, using the unrestricted version of the B3LYP functional, selecting the 6-311G(d) using the IEFPCM with dichloromethane as solvent.

Using the optimized geometries, the diradical index (y_0) was calculated from DFT and CASSCF methods. The complete-active-space self-consistent field method (CASSCF) was first used, we performed two different calculations, selecting 2 electrons and 2 orbitals and 12 electrons and 12 orbitals (CASSCF(2,2) and CASSCF(12,12)). According to Yamaguchi,[S10] the y_0 was extracted from the weight of the doubly excited configuration, calculated by CASSCF methods. We subsequently performed a natural orbital analysis at the BS-U-LC-BLYP/6-311G(d) and BS-CAM-

B3LYP/6-3111G(d) level of theory to obtain the occupancies of the HONO and LUNO (n_{HONO} and n_{LUNO}). y_0 was then calculated from the Yamaguchi equation:

$$y_0 = 1 - \frac{2T_i}{1 + T_i^2}$$

where:

$$T_i = \frac{n_{HONO} - n_{LUNO}}{2}$$

Cartesian coordinates of the optimized geometries are available as .xyz files, free of charge at the Zenodo repository with DOI: 10.5281/zenodo.10981323.

Table S2. Calculated closed-open-shell energy gaps, singlet-triplet energy gaps and expectation value of the total spin (<S^2>) from different functionals for **IF7H**.

	ΔE_{OS-CS} (kcal mol^{-1})	<S^2>$_{OS}$	<S^2>$_T$	$\lvert\Delta E_{S-T}\rvert$ (kcal mol^{-1})	y_0
B3LYP	0.00	0.0000	2.0249	8.36	0.000
CAM-B3LYP	-0.08	0.1402	2.0731	8.34	0.002
LC-BLYP	-1.41	0.5245	2.2137	10.17	0.030
M06-2X	0.00	0.0000	2.0328	11.21	0.000
CASSCF(2,2)	-	-	-	-	0.100[a]
CASSCF(12,12)	-	-	-	-	0.070[a]

[a] Calculated from the weight of the doubly excited state configuration.

Table S3. Calculated closed-open-shell energy gaps, singlet-triplet energy gaps and expectation value of the total spin (<S^2>) from different functionals for **IF5H**.

	ΔE_{OS-CS} (kcal mol^{-1})	<S^2>$_{OS}$	<S^2>$_T$	$\lvert\Delta E_{S-T}\rvert$(kcal mol^{-1})	y_0
B3LYP	-0.01	0.0000	2.0299	13.30	0.000
CAM-B3LYP	-0.05	0.1146	2.0670	12.47	0.001
LC-BLYP	-1.33	0.4579	2.1498	15.09	0.027
M06-2X	-0.01	0.0000	2.0345	15.71	0.000
CASSCF(2,2)	-	-	-	-	0.109[a]
CASSCF(12,12)	-	-	-	-	0.068[a]

[a] Calculated from the weight of the doubly excited state configuration.

*Optimized Geometries of **IF5H** and **IF7H***

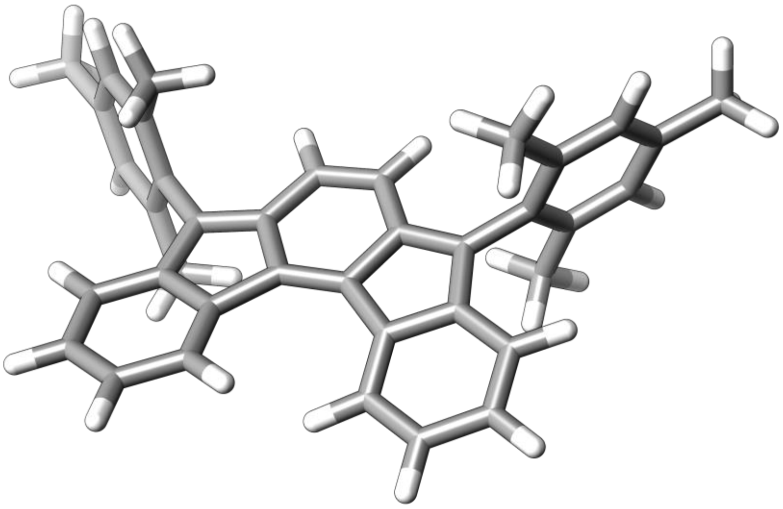

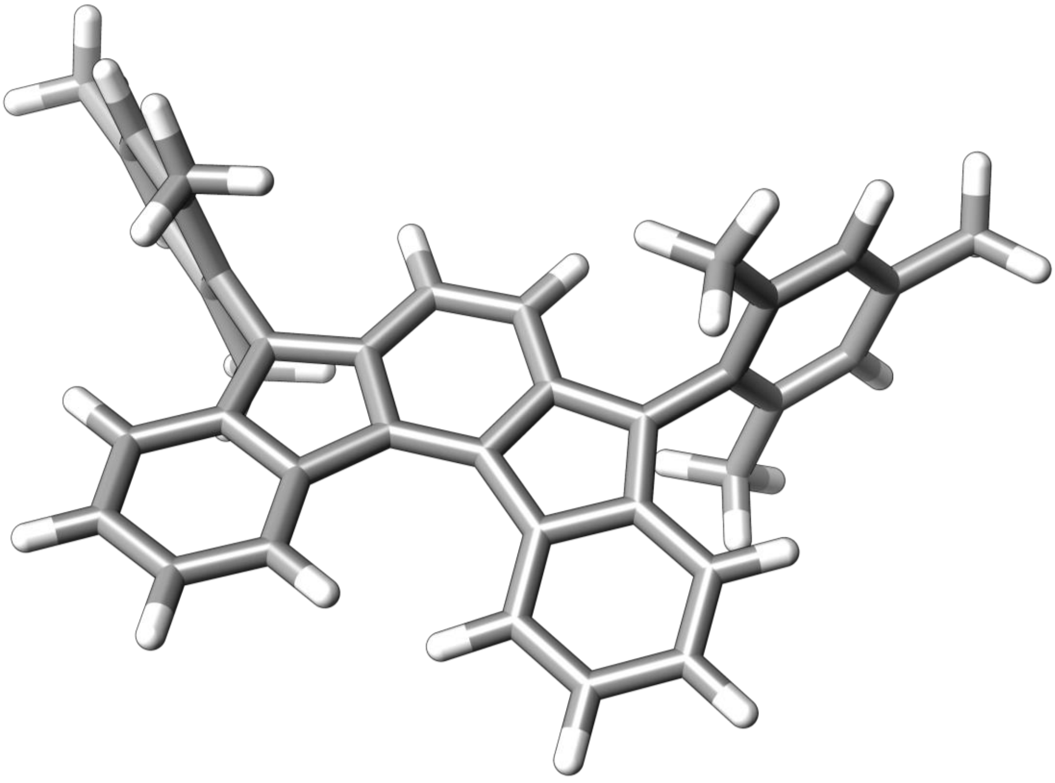

Figure S13. Optimized geometries of compound **IF5H** at its singlet (top, BS-UB3LYP/6-311G(d)) and triplet (bottom, UB3LYP/6311G(d)) states.

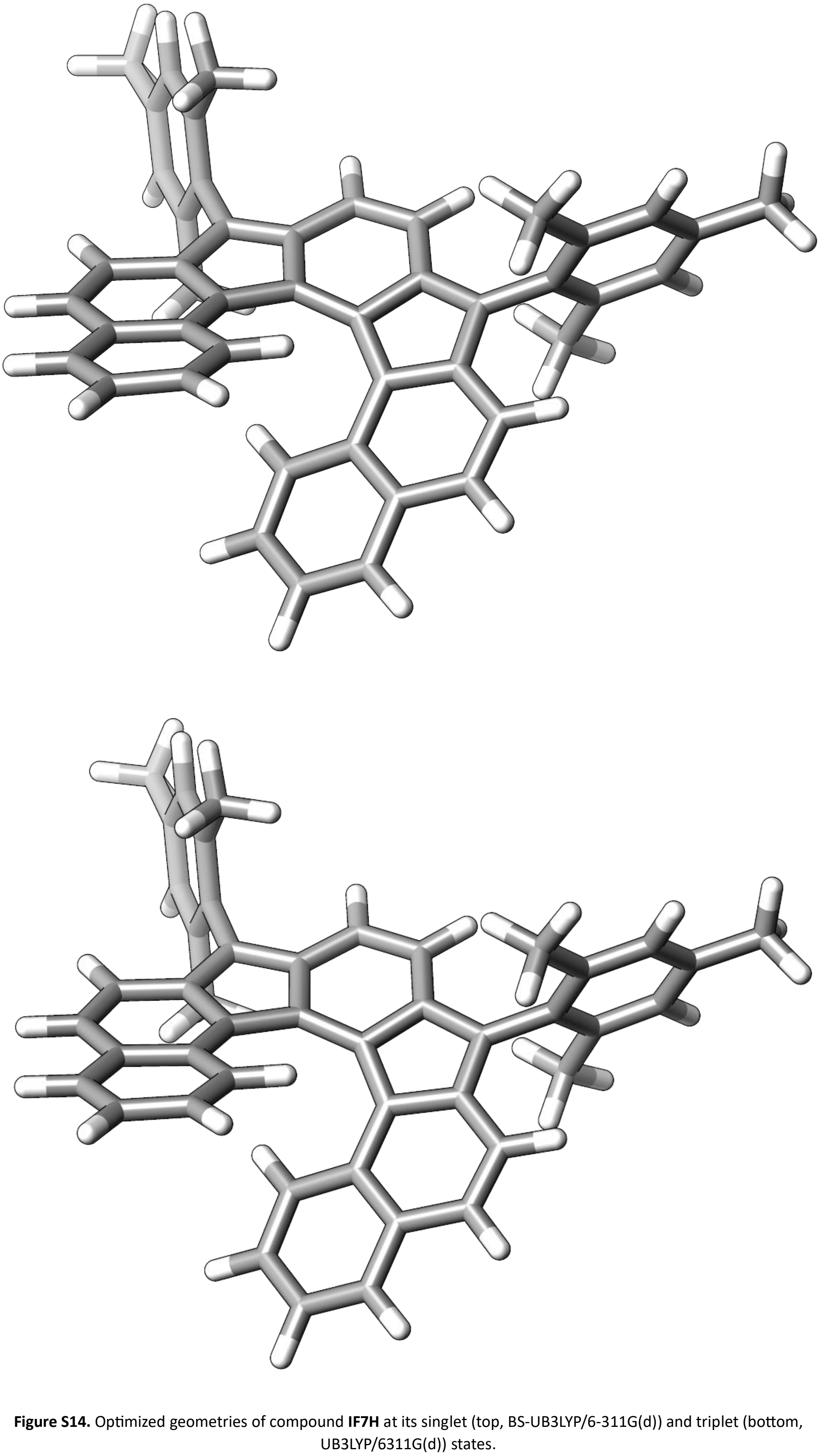

Figure S14. Optimized geometries of compound **IF7H** at its singlet (top, BS-UB3LYP/6-311G(d)) and triplet (bottom, UB3LYP/6311G(d)) states.

HOMO and LUMO Profiles and Energy Values

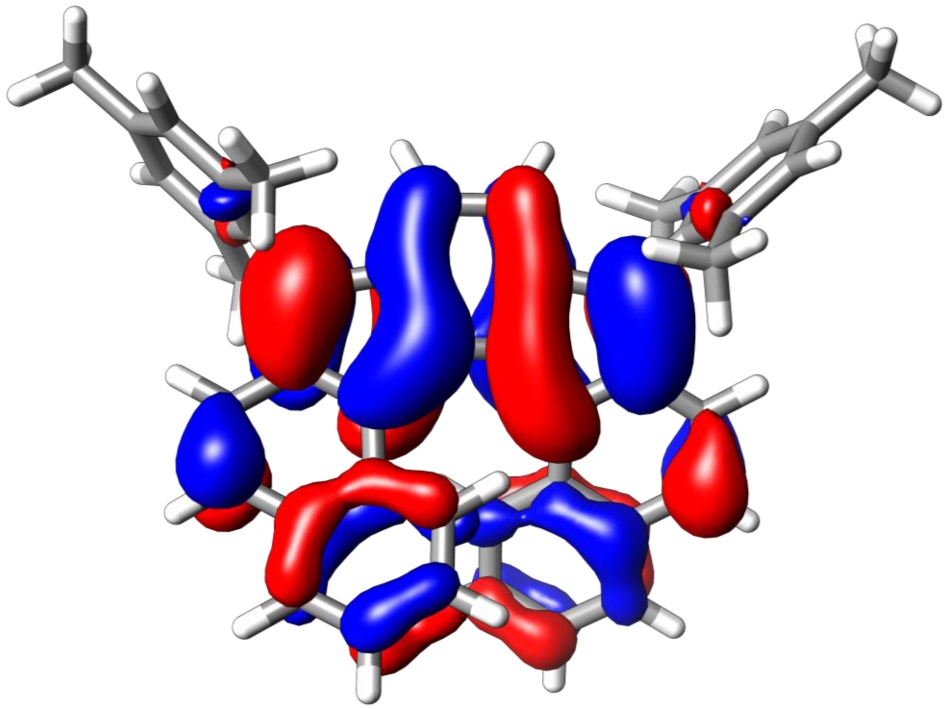

LUMO
–3.20 eV

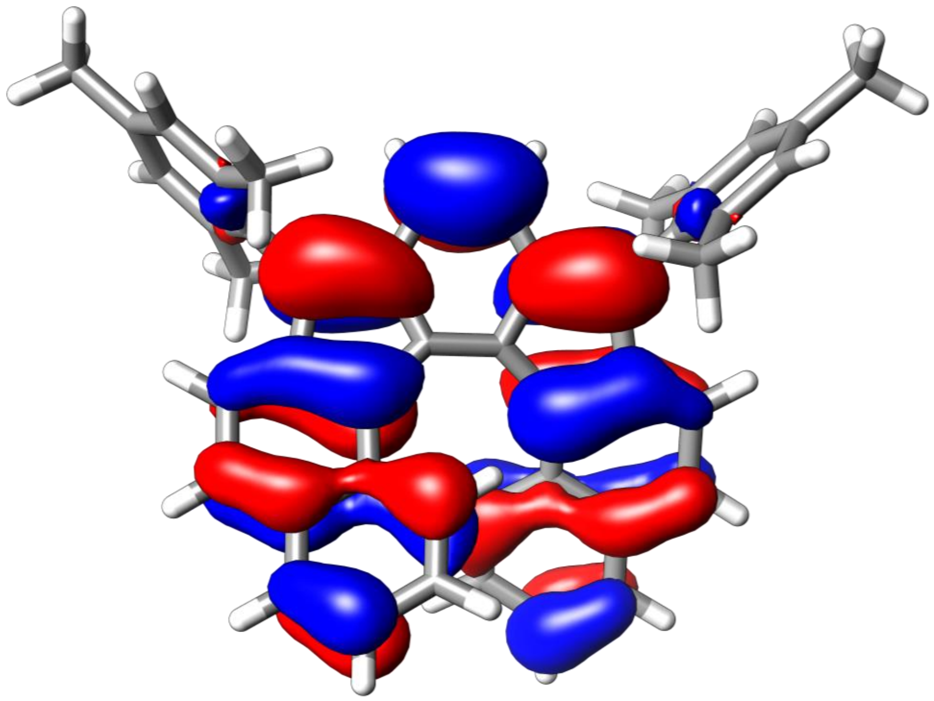

HOMO
–5.14 eV

Figure S15. HOMO and LUMO plots and energy values for **IF7H**.

Spin Densities

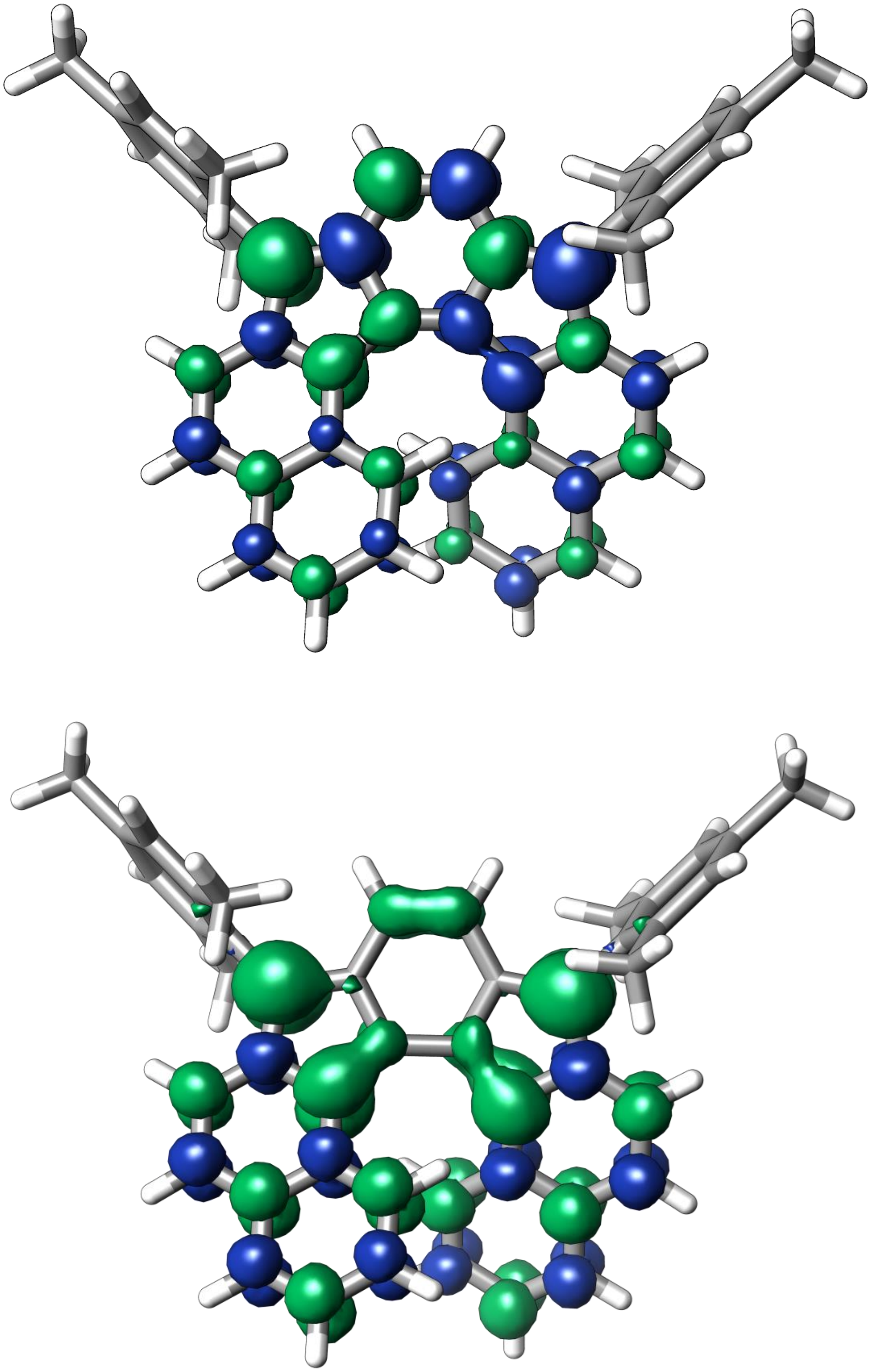

Figure S16. Spin density maps of **IF7H** at its singlet (top) and triplet (bottom) states (isoval=0.004), calculated at the (BS)U-LC-BLYP/6-311G(d).

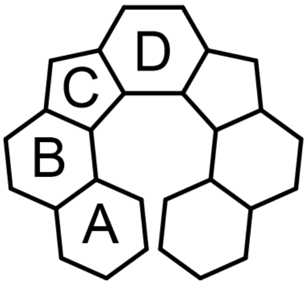

	S		T	
Ring	$NICS(0)_{iso}$	HOMA	$NICS(0)_{iso}$	HOMA
A	-7.28	0.92	-7.26	0.95
B	-5.99	0.94	-3.64	0.89
C	10.18	0.61	2.46	0.79
D	3.27	0.68	-3.07	0.90

Figure S17. Calculated NICS(0) and HOMA values for the different rings of **IF7H** at the singlet and triplet optimized geometries ((BS)U-LC-BLYP/6-311G(d)).

Anisotropy of the Induced Current Density (ACID)

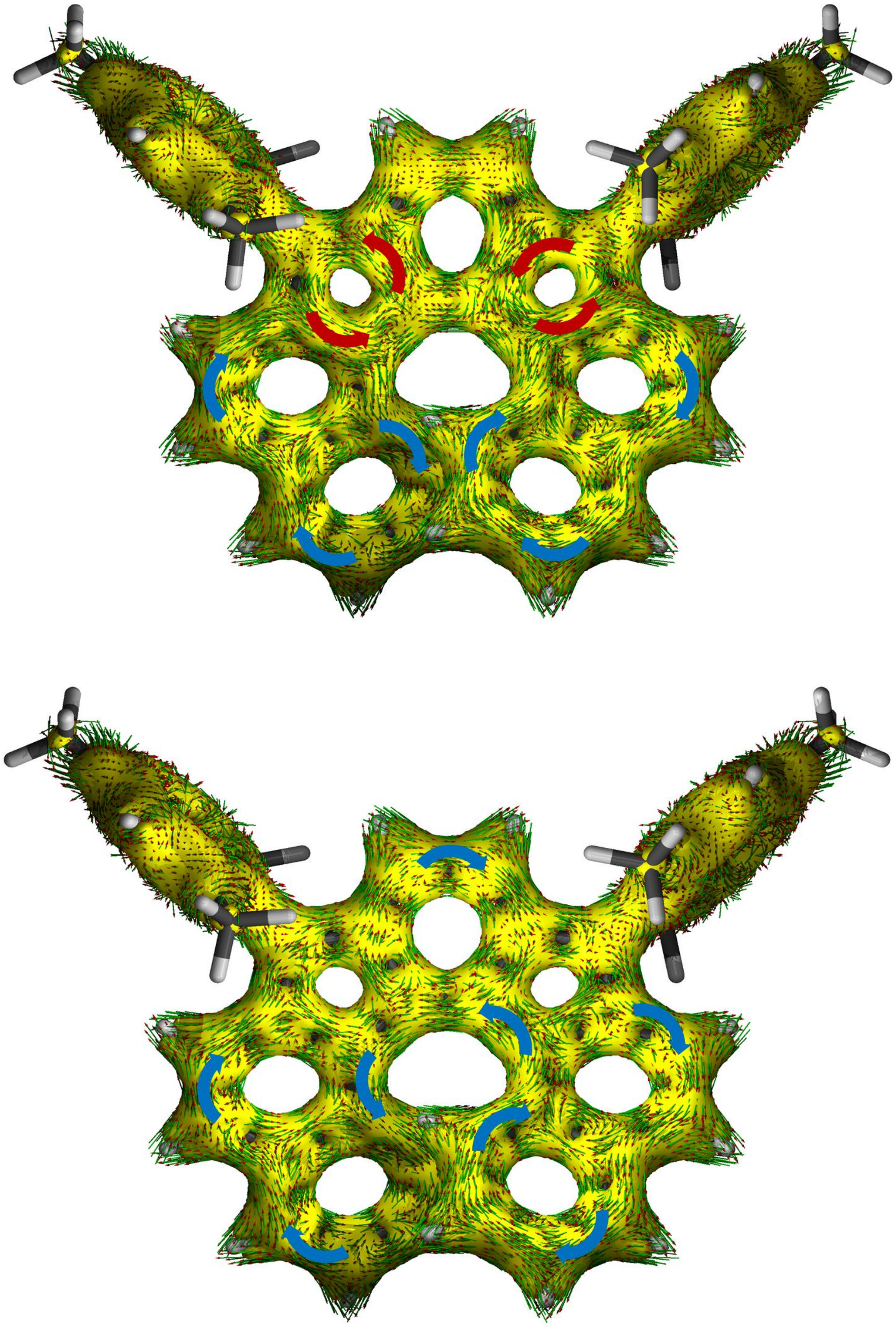

Figure S18. Calculated AICD surfaces and ring currents ((BS)U-LC-BLYP/6-311G(d), isoval = 0.05) of the singlet (top) and triplet (bottom) states of **IF7H**. Magnetic field pointing towards the spectator. Blue arrows: diatropic currents; Red arrows: paratropic currents.

Table S4. Calculated 50 lowest electronic transitions for compound **IF7H**, their energies (in nm) and oscillatory strength (in cgs units). Correction: +0.2 eV.

Transition number	Wavelength / nm	Osc. Strength / cgs	Transition number	Wavelength / nm	Osc. Strength / cgs
1	1651.53	0.0000	26	334.02	0.0000
2	831.84	0.0212	27	326.98	0.0000
3	738.43	0.0000	28	323.97	0.0000
4	649.75	0.0000	29	322.52	0.2267
5	515.56	0.0019	30	322.07	0.0000
6	488.94	0.3306	31	322.05	0.0000
7	450.79	0.0000	32	308.97	0.0000
8	424.71	0.0000	33	301.73	0.0071
9	424.35	0.0000	34	300.51	0.0000
10	420.27	0.0000	35	297.80	0.0173
11	417.89	0.0141	36	295.50	0.0004
12	412.71	0.0275	37	294.22	0.0440
13	404.50	0.0000	38	291.52	0.0000
14	397.59	0.0000	39	289.19	0.0000
15	397.08	0.0000	40	288.65	0.1495
16	395.37	0.0016	41	287.85	0.0000
17	395.23	0.0015	42	282.59	0.0000
18	391.80	0.0000	43	281.70	0.0352
19	378.59	0.0652	44	281.36	0.0174
20	370.52	0.0000	45	280.59	0.0000
21	368.32	0.0000	46	280.08	0.0000
22	367.91	0.1381	47	274.17	0.0000
23	359.15	0.3593	48	272.32	0.0158
24	337.06	0.0000	49	270.46	0.0000
25	335.47	0.0046	50	270.38	0.0000

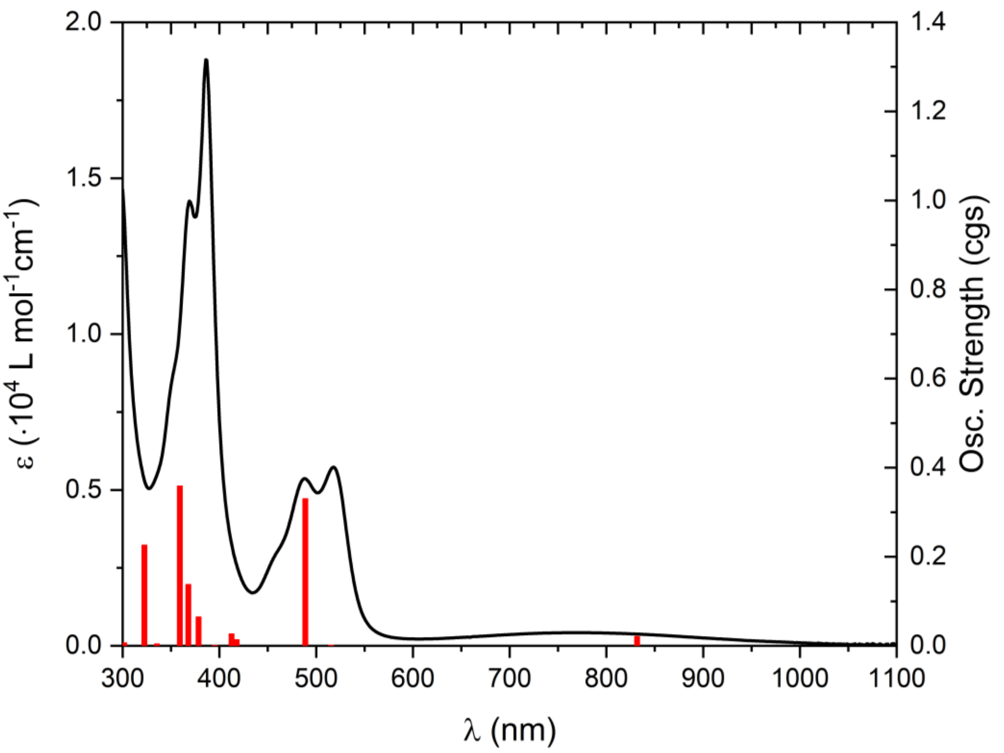

Figure S19. Experimental UV-vis spectrum of compound **IF7H** in chloroform and calculated oscillator strength of the calculated electronic transitions.

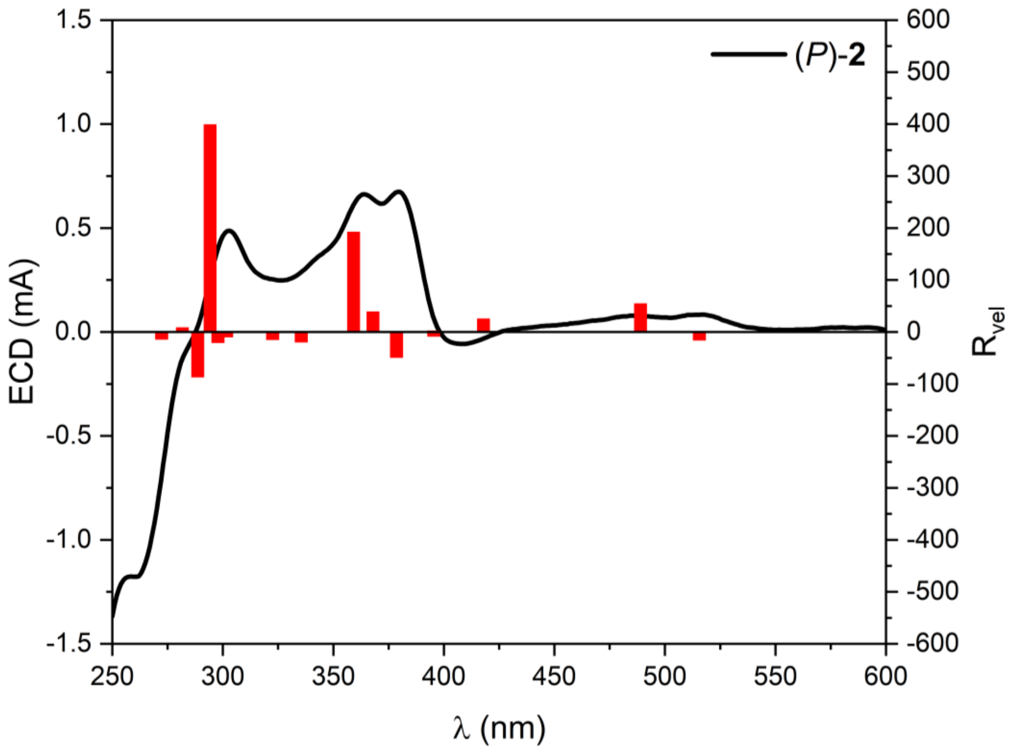

Figure S20. Experimental ECD spectrum of compound (*P*)-**IF7H** in chloroform and calculated rotatory strength of the calculated electronic transitions for compound (*P*)-**IF7H**.

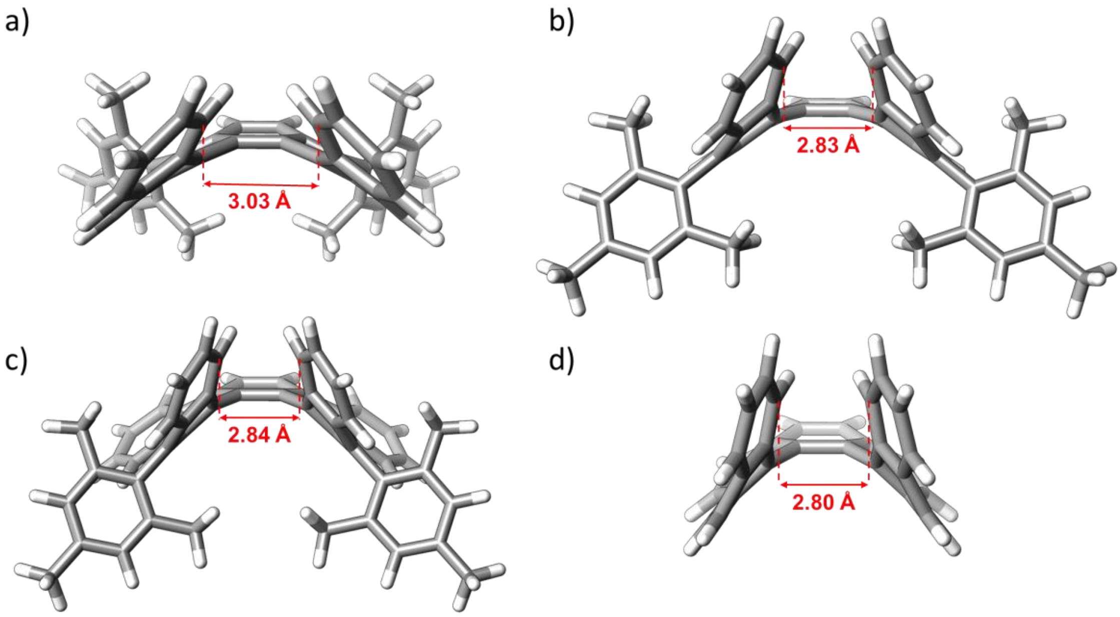

Figure S21. Transition state geometries of: a) **IF7H**; b) dinor[7]helicene; c) 5,14-Diaryldiindeno[2,1-f:1′,2′-j]picene and d) [7]helicene. Distance between the fjord carbon atoms highlighted.

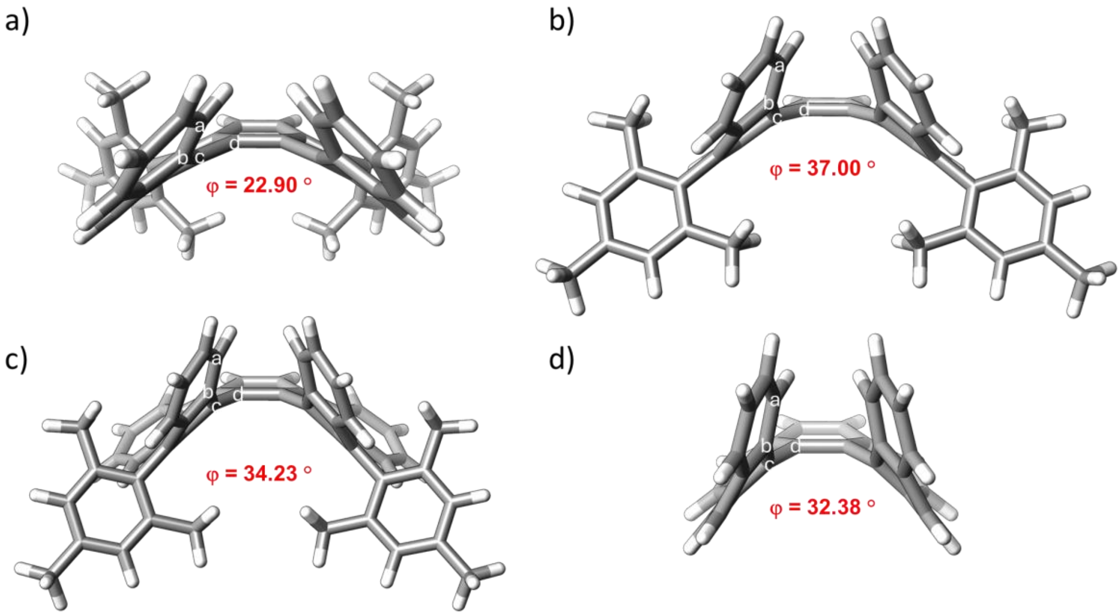

Figure S22. Transition state geometries of: a) **IF7H**; b) dinor[7]helicene; c) 5,14-Diaryldiindeno[2,1-f:1′,2′-j]picene and d) [7]helicene. Dihedral angle highlighted.

Cartesian Coordinates

IF5H singlet state

C	0.00378900	1.39349200	-0.05337400
C	0.03844600	0.68264100	1.24113800
C	-0.03844600	-0.68264100	1.24113800
C	-0.00378900	-1.39349200	-0.05337400
C	0.01208000	-0.67827400	-1.29909500
C	-0.01208000	0.67827400	-1.29909500
C	-0.13104200	-1.73488400	2.27700200
C	-0.06800800	-2.98667200	1.60214700
C	0.01208000	-2.75105600	0.15942500
C	-0.01208000	2.75105600	0.15942500
C	0.06800800	2.98667200	1.60214700
C	0.13104200	1.73488400	2.27700200
C	0.14460500	4.18553300	2.30229700
C	0.30784900	4.14947700	3.69196400
C	0.41509500	2.93018900	4.35537000
C	0.33382100	1.71919600	3.65129600
C	-0.33382100	-1.71919600	3.65129600
C	-0.41509500	-2.93018900	4.35537000
C	-0.30784900	-4.14947700	3.69196400
C	-0.14460500	-4.18553300	2.30229700
C	0.07013900	-3.82184300	-0.86740000
C	-0.07013900	3.82184300	-0.86740000
C	-1.30008000	4.44474200	-1.16545700
C	-1.33356700	5.45617200	-2.12737500
C	-0.18360600	5.87478400	-2.80009400
C	1.02244300	5.24555600	-2.48955900
C	1.10262400	4.22721800	-1.53565500
C	-1.10262400	-4.22721800	-1.53565500
C	-1.02244300	-5.24555600	-2.48955900

C	0.18360600	-5.87478400	-2.80009400
C	1.33356700	-5.45617200	-2.12737500
C	1.30008000	-4.44474200	-1.16545700
C	-2.57513600	4.01792900	-0.47610900
C	2.43685400	3.58337600	-1.23626400
C	-0.24140100	6.99359400	-3.81208800
C	2.57513600	-4.01792900	-0.47610900
C	-2.43685400	-3.58337600	-1.23626400
C	0.24140100	-6.99359400	-3.81208800
H	0.02712000	-1.23880000	-2.22779700
H	-0.02712000	1.23880000	-2.22779700
H	0.09447200	5.13400300	1.77741600
H	0.36948100	5.07694300	4.25175800
H	0.57018900	2.91000800	5.42881800
H	0.46597600	0.79499300	4.19444200
H	-0.46597600	-0.79499300	4.19444200
H	-0.57018900	-2.91000800	5.42881800
H	-0.36948100	-5.07694300	4.25175800
H	-0.09447200	-5.13400300	1.77741600
H	-2.28540300	5.92716800	-2.35955400
H	1.92971200	5.55526100	-3.00183300
H	-1.92971200	-5.55526100	-3.00183300
H	2.28540300	-5.92716800	-2.35955400
H	-2.51585500	4.15547000	0.60748500
H	-3.42830200	4.59406000	-0.83868200
H	-2.78838900	2.95871500	-0.64682700
H	3.24520900	4.10218800	-1.75475800
H	2.66107900	3.59657100	-0.16605300
H	2.46014200	2.53543700	-1.54951800
H	-1.17872400	6.97932400	-4.37371400
H	-0.17436400	7.97178800	-3.32316900

H	0.58208100	6.93191700	-4.52704300
H	3.42830200	-4.59406000	-0.83868200
H	2.78838900	-2.95871500	-0.64682700
H	2.51585500	-4.15547000	0.60748500
H	-2.46014200	-2.53543700	-1.54951800
H	-3.24520900	-4.10218800	-1.75475800
H	-2.66107900	-3.59657100	-0.16605300
H	-0.58208100	-6.93191700	-4.52704300
H	1.17872400	-6.97932400	-4.37371400
H	0.17436400	-7.97178800	-3.32316900

No imaginary frequencies

Zero-point correction = 0.576143 (Hartree/Particle)

E(UB3LYP) = -1467.650052 Hartrees

E(UCAM-B3LYP) = -1466.780622 Hartrees

E(ULC-BLYP) = -1463.068984 Hartrees

E(UM06-2X) = -1467.040056 Hartrees

IF5H triplet state

C	-0.00049300	1.38144700	-0.01189700
C	0.03067400	0.69939200	1.25875300
C	-0.03067400	-0.69939200	1.25875300
C	0.00049300	-1.38144700	-0.01189700
C	0.01012200	-0.69830800	-1.22714300
C	-0.01012200	0.69830800	-1.22714300
C	-0.13643300	-1.74907300	2.27985900
C	-0.09803000	-3.02144500	1.60553700
C	-0.01361500	-2.81287700	0.20295100
C	0.01361500	2.81287700	0.20295100
C	0.09803000	3.02144500	1.60553700
C	0.13643300	1.74907300	2.27985900
C	0.17593200	4.22499000	2.33142900
C	0.32662700	4.17636100	3.70898200
C	0.41469300	2.93861800	4.36574000
C	0.32411200	1.73483100	3.66084700
C	-0.32411200	-1.73483100	3.66084700
C	-0.41469300	-2.93861800	4.36574000
C	-0.32662700	-4.17636100	3.70898200
C	-0.17593200	-4.22499000	2.33142900
C	0.03067400	-3.85370400	-0.85092400
C	-0.03067400	3.85370400	-0.85092400
C	-1.26523100	4.22886500	-1.42088700
C	-1.28455200	5.20930600	-2.41531400
C	-0.11742900	5.82990800	-2.86438100
C	1.09411100	5.44045600	-2.29097500
C	1.16065600	4.46644400	-1.29177300
C	-1.16065600	-4.46644400	-1.29177300
C	-1.09411100	-5.44045600	-2.29097500
C	0.11742900	-5.82990800	-2.86438100

C	1.28455200	-5.20930600	-2.41531400
C	1.26523100	-4.22886500	-1.42088700
C	-2.55812100	3.59363000	-0.96421600
C	2.49981300	4.07481000	-0.71116500
C	-0.16758200	6.90861400	-3.91933200
C	2.55812100	-3.59363000	-0.96421600
C	-2.49981300	-4.07481000	-0.71116500
C	0.16758200	-6.90861400	-3.91933200
H	0.02046000	-1.24914400	-2.16175700
H	-0.02046000	1.24914400	-2.16175700
H	0.12980200	5.17648800	1.81149800
H	0.39195800	5.09521500	4.28209900
H	0.56017900	2.91207300	5.44060400
H	0.43268700	0.80861900	4.20604200
H	-0.43268700	-0.80861900	4.20604200
H	-0.56017900	-2.91207300	5.44060400
H	-0.39195800	-5.09521500	4.28209900
H	-0.12980200	-5.17648800	1.81149800
H	-2.23885100	5.49571100	-2.84997200
H	2.01620200	5.90384200	-2.63257400
H	-2.01620200	-5.90384200	-2.63257400
H	2.23885100	-5.49571100	-2.84997200
H	-2.69966200	3.70124400	0.11501600
H	-3.41587800	4.04980300	-1.46173600
H	-2.58035200	2.52113300	-1.17855600
H	3.31472700	4.58163900	-1.23117400
H	2.57133300	4.33191300	0.34969800
H	2.67244200	2.99739200	-0.78581800
H	-0.99657500	6.75349000	-4.61387300
H	-0.30634300	7.89668800	-3.46665500
H	0.75737500	6.94673200	-4.49938400

H	*3.41587800*	*-4.04980300*	*-1.46173600*
H	*2.58035200*	*-2.52113300*	*-1.17855600*
H	*2.69966200*	*-3.70124400*	*0.11501600*
H	*-2.67244200*	*-2.99739200*	*-0.78581800*
H	*-3.31472700*	*-4.58163900*	*-1.23117400*
H	*-2.57133300*	*-4.33191300*	*0.34969800*
H	*-0.75737500*	*-6.94673200*	*-4.49938400*
H	*0.99657500*	*-6.75349000*	*-4.61387300*
H	*0.30634300*	*-7.89668800*	*-3.46665500*

No imaginary frequencies

Zero-point correction = 0.574224 (Hartree/Particle)

E(UB3LYP) = -1467.626943 Hartrees

E(UCAM-B3LYP) = -1466.759938 Hartrees

E(ULC-BLYP) = -1463.048134 Hartrees

E(UM06-2X) = -1467.013105 Hartrees

IF7H singlet state

C	0.03343100	1.38823400	-0.98970700
C	0.13181900	0.67248300	0.30393100
C	-0.13181900	-0.67248300	0.30393100
C	-0.03343100	-1.38823400	-0.98970700
C	0.02080000	-0.67838500	-2.23703000
C	-0.02080000	0.67838500	-2.23703000
C	-0.39628700	-1.71259100	1.32162300
C	-0.20482000	-2.95200000	0.68582300
C	0.01324400	-2.73925100	-0.75011000
C	-0.01324400	2.73925100	-0.75011000
C	0.20482000	2.95200000	0.68582300
C	0.39628700	1.71259100	1.32162300
C	0.33741400	4.16572700	1.38330900
C	0.73141700	4.13769300	2.70360400
C	1.08721200	2.92009000	3.33773100
C	0.95994800	1.67247500	2.62528000
C	-0.95994800	-1.67247500	2.62528000
C	-1.08721200	-2.92009000	3.33773100
C	-0.73141700	-4.13769300	2.70360400
C	-0.33741400	-4.16572700	1.38330900
C	1.62891100	2.91347400	4.65178300
C	2.08840400	1.75326300	5.22388500
C	2.03710300	0.54112800	4.49421100
C	1.48631500	0.50133300	3.23681500
C	-1.48631500	-0.50133300	3.23681500
C	-2.03710300	-0.54112800	4.49421100
C	-2.08840400	-1.75326300	5.22388500
C	-1.62891100	-2.91347400	4.65178300
C	0.19455200	-3.82609500	-1.74444000
C	-0.19455200	3.82609500	-1.74444000

C	-1.46123000	4.42589000	-1.90498600
C	-1.61402600	5.45141300	-2.83975000
C	-0.54732000	5.90700800	-3.61753500
C	0.69731900	5.30125800	-3.44156400
C	0.89612400	4.26996900	-2.51938100
C	1.46123000	-4.42589000	-1.90498600
C	1.61402600	-5.45141300	-2.83975000
C	0.54732000	-5.90700800	-3.61753500
C	-0.69731900	-5.30125800	-3.44156400
C	-0.89612400	-4.26996900	-2.51938100
C	-2.64986200	3.95756900	-1.09835600
C	2.26930200	3.65727100	-2.36530600
C	-0.73141700	7.03997700	-4.59809400
C	-2.26930200	-3.65727100	-2.36530600
C	2.64986200	-3.95756900	-1.09835600
C	0.73141700	-7.03997700	-4.59809400
H	0.05114400	-1.24078600	-3.16402500
H	-0.05114400	1.24078600	-3.16402500
H	0.14740100	5.10856600	0.88121500
H	0.82895700	5.06327700	3.26276300
H	-0.82895700	-5.06327700	3.26276300
H	-0.14740100	-5.10856600	0.88121500
H	1.69909200	3.85504100	5.18830100
H	2.51410100	1.76551300	6.22176000
H	2.44164100	-0.36544300	4.93250200
H	1.47041300	-0.43190900	2.69009300
H	-1.47041300	0.43190900	2.69009300
H	-2.44164100	0.36544300	4.93250200
H	-2.51410100	-1.76551300	6.22176000
H	-1.69909200	-3.85504100	5.18830100
H	-2.59436800	5.90377600	-2.96614000

H	1.54129500	5.64115700	-4.03627400
H	2.59436800	-5.90377600	-2.96614000
H	-1.54129500	-5.64115700	-4.03627400
H	-2.48526300	4.08036600	-0.02369500
H	-3.54852100	4.51759600	-1.36295100
H	-2.85415700	2.89592400	-1.26489600
H	3.00964000	4.20945800	-2.94703800
H	2.59601900	3.65463200	-1.32180200
H	2.29037400	2.61719700	-2.70367300
H	-1.72140200	7.01449400	-5.06009400
H	-0.63319300	8.01157200	-4.10135000
H	0.01467100	7.00700000	-5.39512300
H	-3.00964000	-4.20945800	-2.94703800
H	-2.59601900	-3.65463200	-1.32180200
H	-2.29037400	-2.61719700	-2.70367300
H	2.48526300	-4.08036600	-0.02369500
H	3.54852100	-4.51759600	-1.36295100
H	2.85415700	-2.89592400	-1.26489600
H	1.72140200	-7.01449400	-5.06009400
H	0.63319300	-8.01157200	-4.10135000
H	-0.01467100	-7.00700000	-5.39512300

No imaginary frequencies

Zero-point correction = 0.668907 (Hartree/Particle)

E(UB3LYP) = -1774.977168 Hartrees

E(UCAM-B3LYP) = -1773.931775 Hartrees

E(ULC-BLYP) = -1769.448689 Hartrees

E(UM06-2X) = -1774.256079 Hartrees

IF7H triplet state

C	0.71097700	1.18546500	-0.93311500
C	0.44017400	0.54520500	0.34145000
C	-0.44017400	-0.54520500	0.34145000
C	-0.71097700	-1.18546500	-0.93311500
C	-0.32955100	-0.61710300	-2.14593000
C	0.32955100	0.61710300	-2.14593000
C	-1.21417900	-1.29274300	1.33206200
C	-1.72133700	-2.46541800	0.68413700
C	-1.43696600	-2.40705500	-0.70063700
C	1.43696600	2.40705500	-0.70063700
C	1.72133700	2.46541800	0.68413700
C	1.21417900	1.29274300	1.33206200
C	2.46839800	3.43750200	1.40367900
C	2.77909600	3.20813500	2.71313500
C	2.44371600	1.96877500	3.34971400
C	1.68232200	0.97289900	2.64203600
C	-1.68232200	-0.97289900	2.64203600
C	-2.44371600	-1.96877500	3.34971400
C	-2.77909600	-3.20813500	2.71313500
C	-2.46839800	-3.43750200	1.40367900
C	2.90923600	1.68003800	4.65125000
C	2.70077700	0.44299800	5.22797700
C	2.02179000	-0.55937300	4.50904000
C	1.51810400	-0.29713200	3.25270900
C	-1.51810400	0.29713200	3.25270900
C	-2.02179000	0.55937300	4.50904000
C	-2.70077700	-0.44299800	5.22797700
C	-2.90923600	-1.68003800	4.65125000
C	-1.83861700	-3.39485100	-1.73095400
C	1.83861700	3.39485100	-1.73095400

C	1.04571700	4.53899600	-1.95601100
C	1.43696600	5.45507600	-2.93522800
C	2.59261600	5.27416500	-3.69690400
C	3.36288900	4.13445900	-3.45862200
C	3.00967100	3.19162700	-2.49044400
C	-1.04571700	-4.53899600	-1.95601100
C	-1.43696600	-5.45507600	-2.93522800
C	-2.59261600	-5.27416500	-3.69690400
C	-3.36288900	-4.13445900	-3.45862200
C	-3.00967100	-3.19162700	-2.49044400
C	-0.21903500	4.77716000	-1.16399100
C	3.88644500	1.98197800	-2.26288700
C	3.01239000	6.29399300	-4.72784200
C	-3.88644500	-1.98197800	-2.26288700
C	0.21903500	-4.77716000	-1.16399100
C	-3.01239000	-6.29399300	-4.72784200
H	-0.57381800	-1.11086000	-3.08049600
H	0.57381800	1.11086000	-3.08049600
H	2.79540600	4.34329200	0.90361300
H	3.34322500	3.94210000	3.28034400
H	-3.34322500	-3.94210000	3.28034400
H	-2.79540600	-4.34329200	0.90361300
H	3.46475100	2.44374500	5.18738700
H	3.07837700	0.23535000	6.22372800
H	1.89221400	-1.54389700	4.94616100
H	1.01068500	-1.08205700	2.70894800
H	-1.01068500	1.08205700	2.70894800
H	-1.89221400	1.54389700	4.94616100
H	-3.07837700	-0.23535000	6.22372800
H	-3.46475100	-2.44374500	5.18738700
H	0.81817100	6.33157200	-3.10950900

H	4.26658900	3.97359100	-4.04096300
H	-0.81817100	-6.33157200	-3.10950900
H	-4.26658900	-3.97359100	-4.04096300
H	-0.01070800	4.90288700	-0.09736700
H	-0.73445300	5.67499900	-1.50988400
H	-0.91346800	3.93661200	-1.25130500
H	4.77109800	2.01429400	-2.90135800
H	4.22447300	1.92077900	-1.22444500
H	3.35575900	1.04952800	-2.47560600
H	2.15225500	6.82822900	-5.13796100
H	3.68090700	7.04413900	-4.29100600
H	3.54934900	5.82902200	-5.55803200
H	-4.77109800	-2.01429400	-2.90135800
H	-4.22447300	-1.92077900	-1.22444500
H	-3.35575900	-1.04952800	-2.47560600
H	0.01070800	-4.90288700	-0.09736700
H	0.73445300	-5.67499900	-1.50988400
H	0.91346800	-3.93661200	-1.25130500
H	-2.15225500	-6.82822900	-5.13796100
H	-3.68090700	-7.04413900	-4.29100600
H	-3.54934900	-5.82902200	-5.55803200

No imaginary frequencies

Zero-point correction = 0.667425 (Hartree/Particle)

E(UB3LYP) = -1774.962361 Hartrees

E(UCAM-B3LYP) = -1773.917895 Hartrees

E(ULC-BLYP) = -1769.434840 Hartrees

E(UM06-2X) = -1774.236729 Hartrees

IF7H TS

C	0.70379000	0.88821800	1.35281000
C	-0.62826700	0.70464300	0.69319600
C	-0.62826700	0.70464300	-0.69319600
C	0.70379000	0.88821800	-1.35281000
C	1.84687800	1.43458100	-0.67440700
C	1.84687800	1.43458100	0.67440700
C	-1.50053100	0.14608100	-1.78521000
C	-0.62441000	-0.27994600	-2.80148100
C	0.70881600	0.26257500	-2.56652400
C	0.70881600	0.26257500	2.56652400
C	-0.62441000	-0.27994600	2.80148100
C	-1.50053100	0.14608100	1.78521000
C	-1.04221200	-1.09456800	3.86984200
C	-2.37022000	-1.43521100	3.97150100
C	-3.32758900	-0.82557400	3.12386000
C	-2.89657400	0.05962500	2.06454600
C	-2.89657400	0.05962500	-2.06454600
C	-3.32758900	-0.82557400	-3.12386000
C	-2.37022000	-1.43521100	-3.97150100
C	-1.04221200	-1.09456800	-3.86984200
C	-4.71539900	-0.96956500	3.38695000
C	-5.64528900	-0.18332000	2.75127900
C	-5.20899900	0.81263300	1.84941000
C	-3.87941700	0.92133500	1.51749000
C	-3.87941700	0.92133500	-1.51749000
C	-5.20899900	0.81263300	-1.84941000
C	-5.64528900	-0.18332000	-2.75127900
C	-4.71539900	-0.96956500	-3.38695000
C	1.85556700	0.11610500	-3.49976900
C	1.85556700	0.11610500	3.49976900

C	2.11717200	1.11637200	4.45740800
C	3.19632900	0.95448100	5.32995700
C	4.02104200	-0.17072600	5.28499800
C	3.74349400	-1.14921200	4.32856700
C	2.67572700	-1.02851000	3.43633700
C	2.67572700	-1.02851000	-3.43633700
C	3.74349400	-1.14921200	-4.32856700
C	4.02104200	-0.17072600	-5.28499800
C	3.19632900	0.95448100	-5.32995700
C	2.11717200	1.11637200	-4.45740800
C	1.25539000	2.35461900	4.54876000
C	2.42297800	-2.10897800	2.41104700
C	5.16182900	-0.33871600	6.25946800
C	1.25539000	2.35461900	-4.54876000
C	2.42297800	-2.10897800	-2.41104700
C	5.16182900	-0.33871600	-6.25946800
H	2.71385700	1.73658500	-1.25160100
H	2.71385700	1.73658500	1.25160100
H	-0.31594200	-1.45349700	4.59129200
H	-2.71171000	-2.10083300	4.75806400
H	-2.71171000	-2.10083300	-4.75806400
H	-0.31594200	-1.45349700	-4.59129200
H	-5.02518600	-1.67774000	4.14970100
H	-6.70045000	-0.28304900	2.98288800
H	-5.92648000	1.51128700	1.43214700
H	-3.54857500	1.74006600	0.90080000
H	-3.54857500	1.74006600	-0.90080000
H	-5.92648000	1.51128700	-1.43214700
H	-6.70045000	-0.28304900	-2.98288800
H	-5.02518600	-1.67774000	-4.14970100
H	3.39631700	1.72973800	6.06516600

H	4.37713600	-2.03061400	4.27158900
H	4.37713600	-2.03061400	-4.27158900
H	3.39631700	1.72973800	-6.06516600
H	0.19853600	2.10313100	4.67524500
H	1.55453700	2.97742800	5.39374100
H	1.32667500	2.96460300	3.64346600
H	1.42267300	-2.54009900	2.51322700
H	2.49322800	-1.71793900	1.39186700
H	3.14657800	-2.92017700	2.50939700
H	5.55545200	0.62599600	6.58783800
H	4.83820000	-0.87833900	7.15642200
H	5.98452900	-0.90819500	5.82061200
H	1.55453700	2.97742800	-5.39374100
H	0.19853600	2.10313100	-4.67524500
H	1.32667500	2.96460300	-3.64346600
H	1.42267300	-2.54009900	-2.51322700
H	3.14657800	-2.92017700	-2.50939700
H	2.49322800	-1.71793900	-1.39186700
H	5.98452900	-0.90819500	-5.82061200
H	4.83820000	-0.87833900	-7.15642200
H	5.55545200	0.62599600	-6.58783800

Imaginary frequencies: -64.26 cm^{-1}

Zero-point correction = 0.668907 (Hartree/Particle)

E(UB3LYP) = -1774.936136 Hartrees

10.References

[S1] Cadart, T.; Nečas, D.; Kaiser, R. P.; Favereau, L.; Císařová, I.; Gyepes, R.; Hodačová, J.; Kalíková, K.; Bednárová, L.; Crassous, J.; Kotora, M. *Chem. Eur. J.* **2021**, *27*, 11279–11284.

[S2] Sheldrick, G. M. *Acta Cryst.* **2015**, *A71*, 3.

[S3] (a) Sheldrick, G. M. *Acta Cryst*. **2008**, *A64*, 112; (b) Sheldrick, G. M. *Acta Cryst.* **2015**, *C71*, 3.

[S4] Farrugia, L. J. *J. Appl. Cryst.* **2012**, *45*, 849.

[S5] Elgrishi, N.; Rountree, K. J.; McCarthy, B. D.; Rountree, E. S.; Eisenhart, T. T.; Dempsey, J. L. A Practical Beginner's Guide to Cyclic Voltammetry. *J. Chem. Educ.* **2018**, 95, 197–206.

[S6] Bleaney, B.; Bowers, K. D. *Proc. R. Soc. London, Ser. A,* **1952***,* 214, 451-465.

[S7] Gaussian 09, Revision B.01, Frisch, M. J.; Trucks, G. W.; Schlegel, H. B.; Scuseria, G. E.; Robb, M. A.; Cheeseman, J. R.; Scalmani, G.; Barone, V.; Petersson, G. A.; Nakatsuji, H.; Li, X.; Caricato, M.; Marenich, A. V.; Bloino, J.; Janesko, B. G.; Gomperts, R.; Mennucci, B.; Hratchian, H. P.; Ortiz, J. V.; Izmaylov, A. F.; Sonnenberg, J. L.; Williams-Young, D.; Ding, F.; Lipparini, F.; Egidi, F.; Goings, J.; Peng, B.; Petrone, A.; Henderson, T.; Ranasinghe, D.; Zakrzewski, V. G.; Gao, J.; Rega, N.; Zheng, G.; Liang, W.; Hada, M.; Ehara, M.; Toyota, K.; Fukuda, R.; Hasegawa, J.; Ishida, M.; Nakajima, T.; Honda, Y.; Kitao, O.; Nakai, H.; Vreven, T.; Throssell, K.; Montgomery, J. A., Jr.; Peralta, J. E.; Ogliaro, F.; Bearpark, M. J.; Heyd, J. J.; Brothers, E. N.; Kudin, K. N.; Staroverov, V. N.; Keith, T. A.; Kobayashi, R.; Normand, J.; Raghavachari, K.; Rendell, A. P.; Burant, J. C.; Iyengar, S. S.; Tomasi, J.; Cossi, M.; Millam, J. M.; Klene, M.; Adamo, C.; Cammi, R.; Ochterski, J. W.; Martin, R. L.; Morokuma, K.; Farkas, O.; Foresman, J. B.; Fox, D. J. Gaussian, Inc., Wallingford CT, 2016.

[S8] Gaussian 16, Revision C.01, Frisch, M. J.; Trucks, G. W.; Schlegel, H. B.; Scuseria, G. E.; Robb, M. A.; Cheeseman, J. R.; Scalmani, G.; Barone, V.; Petersson, G. A.; Nakatsuji, H.; Li, X.; Caricato, M.; Marenich, A. V.; Bloino, J.; Janesko, B. G.; Gomperts, R.; Mennucci, B.; Hratchian, H. P.; Ortiz, J. V.; Izmaylov, A. F.; Sonnenberg, J. L.; Williams-Young, D.; Ding, F.; Lipparini, F.; Egidi, F.; Goings, J.; Peng, B.; Petrone, A.; Henderson, T.; Ranasinghe, D.; Zakrzewski, V. G.; Gao, J.; Rega, N.; Zheng, G.; Liang, W.; Hada, M.; Ehara, M.; Toyota, K.; Fukuda, R.; Hasegawa, J.; Ishida, M.; Nakajima, T.; Honda, Y.; Kitao, O.; Nakai, H.; Vreven, T.; Throssell, K.; Montgomery, J. A., Jr.; Peralta, J. E.; Ogliaro, F.; Bearpark, M. J.; Heyd, J. J.; Brothers, E. N.; Kudin, K. N.; Staroverov, V. N.; Keith, T. A.; Kobayashi, R.; Normand, J.; Raghavachari, K.; Rendell, A. P.; Burant, J. C.; Iyengar, S. S.; Tomasi, J.; Cossi, M.; Millam, J. M.; Klene, M.; Adamo, C.; Cammi, R.; Ochterski, J. W.; Martin, R. L.; Morokuma, K.; Farkas, O.; Foresman, J. B.; Fox, D. J. Gaussian, Inc., Wallingford CT, 2016.

[S9] a) Neese, F. *WIREs Comput. Mol. Sci.* **2012**, *2*, 73-78; b) Neese, F. *WIREs Comput. Mol. Sci.* **2022**, e1606.

[S10] Yamaguchi, K. *Chem. Phys. Lett.* **1975**, *33*, 330.
